# Melatonin orchestrates mitochondrial fusion dynamics-mediated WNT/β-catenin signaling to promote dopaminergic neuronal differentiation of human iPS and nerve regeneration in a MPTP-induced mouse model of Parkinson’s disease

**DOI:** 10.1038/s41420-025-02906-x

**Published:** 2025-12-20

**Authors:** Ping Zhang, Peng Huang, Qiongye Dong, Juan Luo, Guanghui Cui, Xin Guo, Minghua Li, Xia Long, Hongyu Zhang, Wei V. Zheng, Peng Cui

**Affiliations:** 1 Department of Hematology, Peking University Shenzhen Hospital, Shenzhen, Guangdong People’s Republic of China; 2 Intervention and Cell Therapy Center, Peking University Shenzhen Hospital, Shenzhen, Guangdong People’s Republic of China; 3 Neurosurgery, Peking University Shenzhen Hospital, Shenzhen, Guangdong People’s Republic of China; 4 Institute of Precision of Medicine, Peking University Shenzhen Hospital, Shenzhen, Guangdong People’s Republic of China; 5 Central Laboratory, Peking University Shenzhen Hospital, Shenzhen, Guangdong People’s Republic of China

**Keywords:** Induced pluripotent stem cells, Spinal cord injury, Neonatal brain damage

## Abstract

Parkinson’s disease (PD) is a challenging neurodegenerative disorder. Recently, therapy of neural stem cells (NSCs) derived from human induced pluripotent stem cells (hiPSCs) has emerged as a significant advancement in regenerative medicine. Melatonin (MT), acting as a mitochondrial targeting hormone, exhibits neuroprotective properties in neurodegenerative diseases and modulates stem cell differentiation through mitochondrial dynamics. However, the precise mechanism by which MT influences dopaminergic (DA) neuronal differentiation in hiPSCs through regulating mitochondrial dynamics remains unclear. In this study, we developed and optimized a technical protocol for the in vitro functional neuronal differentiation of hiPSCs. Our findings demonstrate that MT enhances the differentiation potential of hiPSCs toward neuroectoderm and significantly improves the efficiency of NSCs differentiation into DA neurons by more than three times within hiPSCs. Using the specific MT receptor inhibitor, Luzindole, we confirmed its inhibitory effect on MT-mediated promotion of neural differentiation. Mechanistically, we propose that MT enhances functional DA neuron differentiation from hiPSCs by activating mitochondrial dynamics-mediated WNT/β-catenin signaling pathways. Additionally, we elucidated the critical role of mitofusin2 (MFN2) in enhancing the directed differentiation of DA neurons from hiPSCs. In vivo studies validated the efficacy of MT-treated hiPSC-derived DA progenitor cells in regenerating tyrosine hydroxylase (TH)-positive DA neurons and improving motor function in a MPTP-induced mouse model of Parkinson’s disease. In conclusion, this study highlights the potential clinical relevance of MT-enhanced differentiation of hiPSCs into DA neurons, offering promising implications for the treatment of PD.

**Figure:**
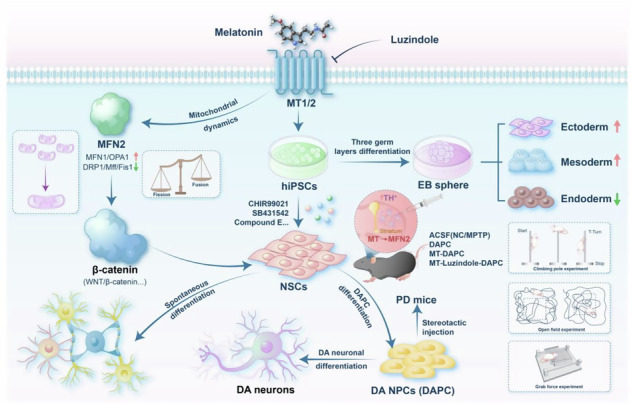
Melatonin orchestrates mitochondrial fusion dynamics-mediated WNT/β-catenin signaling to promote dopaminergic neuronal differentiation of human iPS and nerve regeneration in a MPTP-induced mouse model of Parkinson’s disease.

Melatonin orchestrates mitochondrial fusion dynamics-mediated WNT/β-catenin signaling to promote dopaminergic neuronal differentiation of human iPS and nerve regeneration in a MPTP-induced mouse model of Parkinson’s disease.

## Introduction

Parkinson’s disease (PD) is the second most prevalent systemic neurodegenerative disorder worldwide, characterized by a multifactorial etiology and a range of clinical phenotypes [1]. The hallmark feature of PD involves the degeneration of dopamine (DA) neurons in the substantia nigra pars compacta. In addition to cellular stress and functional impairment, this degeneration largely results in diminished levels of tyrosine hydroxylase (TH) and a subsequent reduction in DA secretion, leading to symptoms such as bradykinesia [2–7]. Melatonin (MT), an indoleamine synthesized in the pineal gland, regulates circadian rhythms and plays vital physiological roles in the central nervous system. It has anti-apoptotic, antioxidant, anti-inflammatory and neuroprotective properties, which are essential for maintaining CNS homeostasis. Notably, declining MT levels are commonly observed in neurodegenerative conditions associated with aging [8, 9]. MT and its metabolites (AFMK and AMK) scavenge free radicals in brain tissues, thereby exerting neuroprotective effects. These actions largely occur through MT receptor 1 A (MT1) and MT receptor 1B (MT2), which are pivotal in mediating MT’s physiological functions [10, 11]. Studies indicate that decreased MT1 and MT2 receptor levels in specific brain regions may contribute to PD pathogenesis. MT modulates PTEN-induced putative kinase 1 (PINK1) expression via the MT2/Akt/NF-κB pathway to inhibit neuronal apoptosis in neurodegenerative diseases [12]. Additionally, MT1 is essential for regulating clock gene expression in striatal neurons, further emphasizing its role in PD [13]. Therefore, PD is considered a neurodegenerative disease resulting from an imbalance between MT and DA. Given this connection, MT can mitigate the loss of DA neurons, thereby preventing a decline of DA levels in PD [14]. Thus, enhancing MT levels to regulate DA levels represents a promising therapeutic strategy for PD treatment.

Human pluripotent stem cells hold a significant promise in regenerative medicine, particularly in cell replacement therapy for PD. Current research has proposed promising protocols capable of reversing DA-related deficits in midbrain dopamine (mDA) neurons, applicable to PD models. However, generating functional mDA neurons suitable for clinical use remains challenging [15–17]. Human induced pluripotent stem cells (hiPSCs) offer a readily accessible and expandable cell source for producing human DA neurons for PD cell-based therapies. Nonetheless, the limited initial survival and functional maturity of transplanted DA neurons in vivo remain substantial obstacles [16, 17]. Studies indicate that the initial survival rates of transplanted mDA neurons are typically below 10%. Furthermore, these neurons express hindbrain and diencephalic markers, which increases the risk of adverse effects post-therapy [18, 19]. Additionally, research has shown that MT enhances cell viability in stem cells and regulates the differentiation of neural stem cells (NSCs) in the embryonic mouse brain at E15.5 [20, 21]. Notably, MT has been demonstrated to improve the engraftment of NSCs in PD mouse models by reducing oxidative stress associated with increased mitochondrial activity. Moreover, combined treatment with NSC transplantation and MT in PD mice effectively restores functional neuronal populations by enhancing mitochondrial function [22]. However, the specific molecular mechanisms through which MT promotes DA neuron differentiation and improves PD models, from hiPSC differentiation to functional DA neurons, remain unclear.

Mitochondria can reshape their morphology through the process of mitochondrial membrane fission and fusion, known as “mitochondrial dynamics.” Mitochondria play important roles in cellular energy production, inter-organelle signal transmission, neuronal axonal transport, and synaptic signaling. They are strictly regulated to adapt to diverse cellular functions by altering their morphology [23, 24]. Studies indicate that dysregulation of mitochondrial dynamics can lead to neurodevelopmental abnormalities. The mitochondrial fusion protein mitofusin2 (MFN2) is essential for neuronal maturation and synapse formation. Conditional knockout of MFN2 has been linked to neurodegenerative conditions, manifesting in reduced cerebellar size and motor impairments [25, 26]. During embryogenesis, WNT signaling orchestrates neurodevelopmental processes, with β-catenin acting as a key mediator in the WNT/β-catenin pathway [27, 28]. In addition, emerging evidence suggests that β-catenin is crucial for maintaining mitochondrial homeostasis under pathological conditions. In models of neurodegenerative diseases, such as 6-hydroxydopamine (6-OHDA)-induced mitochondrial toxicity, activation of WNT/β-catenin signaling has been shown to mitigate neuronal damage [29]. Our previous research demonstrated that MFN2 facilitates NSC differentiation and development by regulating WNT signaling mediated by mitochondrial metabolism [30]. MT, a hormone that targets mitochondria, can permeate the mitochondrial membrane [31, 32]. It enhances mitochondrial fusion and suppresses fission processes, thereby ameliorating damage associated with myocardial and cerebral ischemic diseases [33–35]. Moreover, MT exerts neuroprotective effects by activating β-catenin [28]. However, the precise mechanisms through which MT enhances the differentiation efficiency of functional DA neurons from hiPSCs and its potential therapeutic effects in PD models. Particularly through the regulation of mitochondrial dynamics and the associated WNT signaling pathway, remains to be elucidated.

Numerous studies have demonstrated that MT-regulated mitochondrial dynamics plays an extremely important role in the occurrence and progression of neurodegenerative diseases, highlighting its significant potential for targeted and efficient treatment of PD. By focusing on enhancing the efficiency and functionality of DA neuron differentiation from hiPSCs, this study aims to elucidate the intrinsic regulatory mechanisms via which MT enhances the differentiation efficiency of functional DA neurons and to explore its therapeutic potential in treating PD.

## Results

### MT regulates the differentiation potential of hiPSCs into neuroectoderm and NSCs in vitro

**Fig. 1:**
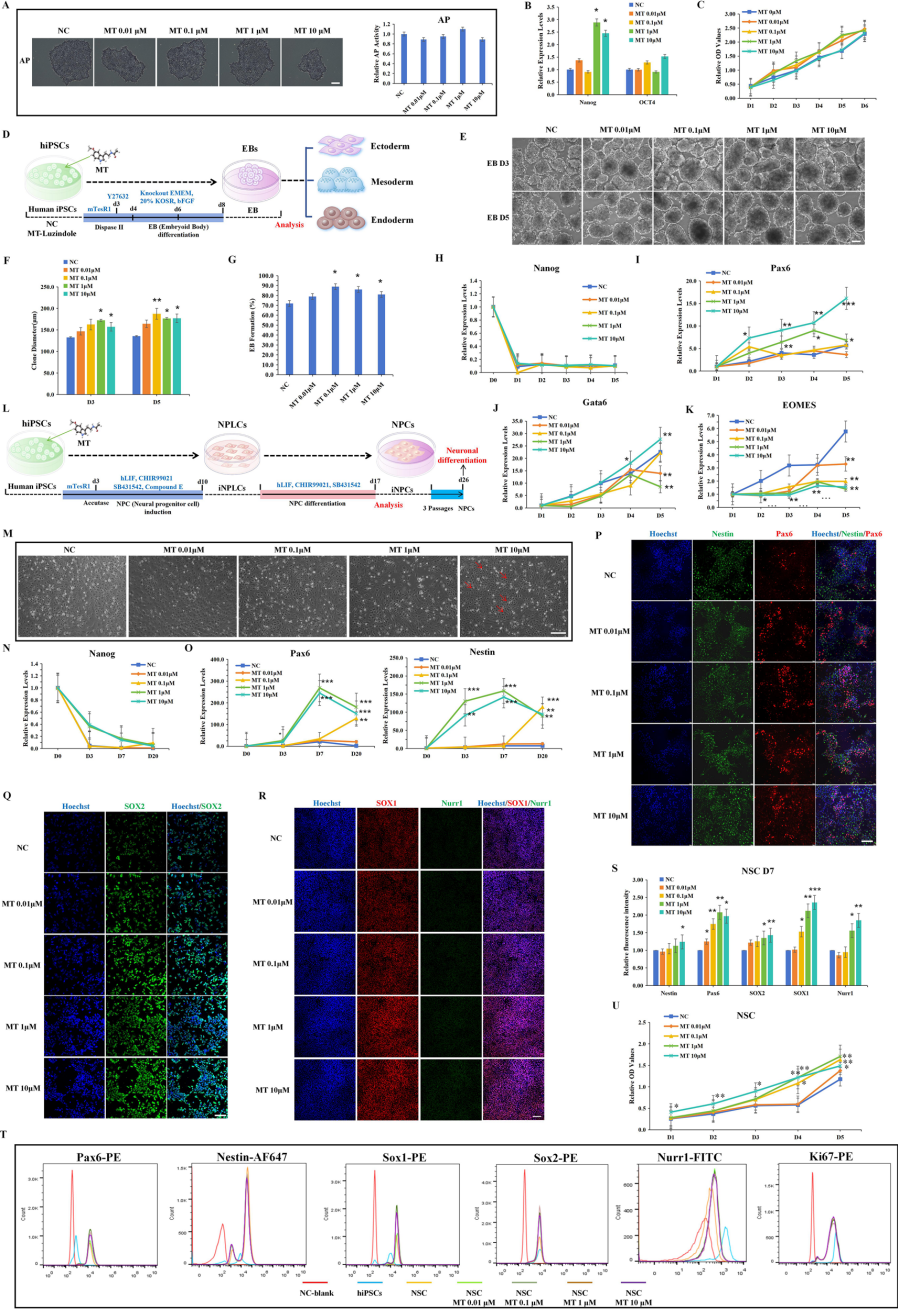
MT regulates the differentiation potential of hiPSCs into neuroectoderm and NSCs in vitro. **A** Alkaline phosphatase staining and relative AP activity statistics. AP staining intensity was measured using the image J software.Scale bars, 20 μm. **B** Expression of pluripotency markers (Nanog and OCT4) in hiPSCs at mRNA level. **C** Relative OD values measured by CCK8 experiments reflecting the self-renewal ability of hiPSCs treated with different concentrations of MT. **D** An optimized protocol of EB random differentiation. **E** The morphology of EB differentiated on days 3 and 5. Scale bars, 50 μm. **F**, **G** The statistics of EB clone diameter and formation efficiency on day 5. **H**–**K** Expression of pluripotency marker (Nanog) and three germ layer markers (Pax6, Gata6, EOMES) was determined by qRT-PCR. **L** An enhanced small molecule induction scheme to differentiate hiPSCs into NSCs. **M** The morphology of differentiated NSC with standard neural rosettes with varying concentrations of MT on day 7. White arrows, neural rosette. Scale bars, 50 μm. **N**, **O** Expression of pluripotency marker (Nanog) and NSC markers (Pax6, Nestin) was determined by qRT-PCR. (**P–R**) Immunofluorescence staining of NSC markers (Pax6, Nestin, SOX1, SOX2) and the midbrain neural marker (Nurr1). Scale bars, 50 μm. **S** The statistics of the relative fluorescence intensity in differentiated NSCs on day 7, and fluorescence intensity data were analyzed using ZEN microscope imaging software. (**T**) Flow cytometry analysis of NSC markers (Pax6, Nestin, SOX2, SOX1) and the midbrain neural marker (Nurr1). **U** Relative OD values measured by CCK8 experiments reflecting the self-renewal ability of NSCs treated with different concentrations of MT. *P < 0.05, **P < 0.01, ***P < 0.001 *vs*. NC.

Previous studies have demonstrated the neuroprotective effects of MT both in vivo and in vitro [9]. However, its impact on the functional differentiation of hiPSCs remains unclear. In order to investigate the effect of MT on hiPSCs differentiation, cells were treated with varying concentrations of MT in vitro. Alkaline phosphatase staining revealed that MT did not significantly affect the pluripotency of hiPSCs after 7 days of treatment (Fig. 1A). Quantitative RT-PCR **(**RT-qPCR) and immunofluorescence analyses, however, showed that higher concentrations (10 μM) of MT increased the expression of pluripotency markers in hiPSCs to a notable extent compared to the negative control (NC) (Figs. 1B, S1A). To assess the self-renewal ability of cells, cell number and size of colonies formed were counted and analyzed through in vitro colony formation assays. This result indicated that MT had no significant impact on cell self-renewal ability (Fig. S1B–D). Consistent results were obtained from CCK8 experiments, confirming MT’s limited effect on hiPSCs self-renewal potential (Fig. 1C).

To further investigate the impact of MT on the differentiation of hiPSCs in vitro, we conducted an Embryoid Body (EB) random differentiation experiment. hiPSCs were treated with varying concentrations of MT over a 7-day period using an optimized protocol (Fig. 1D). Assessment of EB characteristics on days 1-5 revealed that increasing MT concentration significantly enhanced both the efficiency of in vitro EB formation and the clonal size of hiPSCs (Fig. 1E–G). Furthermore, we examined the impact of different concentrations of MT on the differentiation potential of hiPSCs into the three germ layers in vitro. RT-qPCR analysis revealed that under conditions of random differentiation, MT-treated groups with varying concentrations effectively underwent tridermal differentiation. During this process, the expression of the pluripotency gene Nanog significantly decreased as differentiation proceeded (Fig. 1H). Notably, a high concentration of MT (10 μM) significantly enhanced the differentiation potential of neuroectoderm (Pax6) and mesoderm (Gata6) (Fig. 1I, J, Fig. S1E, F), while concurrently inhibiting the differentiation potential of endoderm (EOMES) (Fig. 1K, Fig. S1G). The above results demonstrate that MT has varying effects on the differentiation of hiPSCs into different germ layers and suggest that MT can influence the efficiency and potential of directing hiPSCs toward functional cells across various germ layers.

While NSCs and DA neural progenitor cells are important cell sources for stem cell therapy in neurodegenerative diseases, there is still a need to improve their differentiation efficiency and functional maturity. Since the above experiments indicate that MT enhances the efficiency of hiPSC differentiation into neuroectoderm, it remains to be determined whether MT also improves the differentiation efficiency and functional maturity of hiPSCs into NSCs and DA neurons. To further investigate the effects of MT on the directed differentiation of hiPSCs into neural cells, we employed an enhanced small molecule induction method to differentiate hiPSCs into NSCs (Fig. 1L), incorporating different concentrations of MT in the process. Following a two-step NSC induction process, treatment with higher concentrations of MT (1 μM/10 μM) could lead to more successful and efficient differentiation of hiPSCs into NSCs after 7 days, as demonstrated by NSC morphology with standard neural rosettes (Fig. 1M). RT-qPCR analysis revealed that the expression of NSC markers (Pax6, Nestin) were upregulated significantly in higher concentrations of MT (1 μM/10 μM), indicating an obvious promotion of differentiation into NSC by MT (Fig. 1N, O). Immunofluorescence and flow cytometry results confirmed that high concentrations of MT (1 μM/10 μM) increased the expression of NSC markers Pax6, Nestin, sex determining region Y-box 2 (SOX2), and sex determining region Y-box 1 (SOX1) (Fig. 1P–T). Furthermore, treatment of MT notably increased the expression of the midbrain neural marker (Nurr1) (Fig. 1R–T), suggesting that MT can promote hiPSC differentiation towards midbrain lineage neurons. Additionally, the result of the Cell Counting Kit-8‌ (CCK8) experiment showed that MT significantly promoted the self-renewal potential of differentiated NSCs (Fig. 1U).

### MT promotes the DA neuronal differentiation potential of hiPSCs

**Fig. 2:**
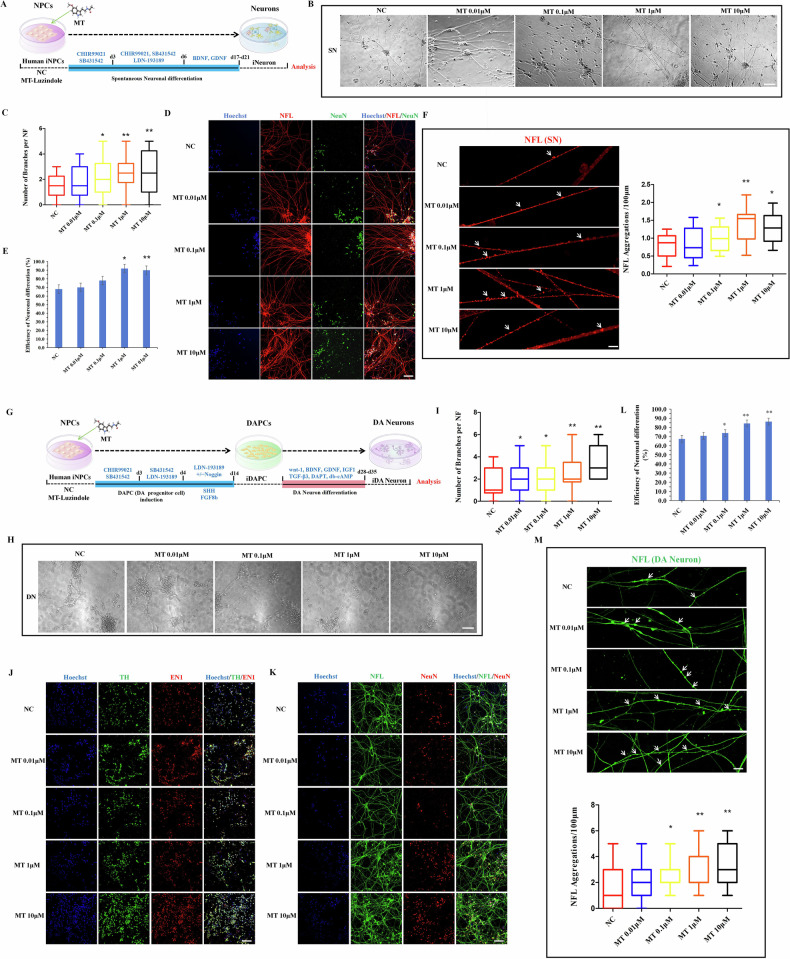
MT promotes DA neuronal differentiation potential of hiPSCs. **A** A small molecule induction scheme to spontaneously differentiate hiPSCs into neurons. **B** The morphology of differentiated neurons with varying concentrations of MT. Scale bars, 20 μm. (**C**) The statistics of the number of branches per NF in spontaneously differentiated neurons. **D** **E** Immunofluorescence staining of neuron markers (NFL, NeuN) and the differentiation efficiency statistics. The efficiency of neuronal differentiation is demonstrated by the positive proportion of neuronal markers (such as the ratio of cells stained positively for NeuN in the cell bodies to the original inoculated cells. Scale bars, 50 μm. **F** Immunofluorescence staining and quantitative analysis of neuronal aggregations. White arrows, neuronal aggregation. Scale bars, 10 μm. **G** A small molecule-induced three-step scheme to differentiate NSCs into DA neurons. **H** The morphology of differentiated DA neurons with varying concentrations of MT. Scale bars, 20 μm. **I** The statistics of the number of branches per NF in directed differentiated DA neurons. **J**–**L** Immunofluorescence staining of DA neuron markers (TH, EN1, NFL, NeuN) and the differentiation efficiency statistics. Scale bars, 50 μm. **M** Immunofluorescence staining and quantitative analysis of DA neuronal aggregations. White arrows, neuronal aggregation. Scale bars, 10 μm. *P < 0.05, **P < 0.01 *vs*. NC.

To further investigate whether MT promotes the differentiation of NSCs into functional neurons, we initially induced NSCs to undergo spontaneous differentiation into neurons using a two-step small molecule induction method (Fig. 2A). The results demonstrated that MT significantly enhances the potential of differentiation of NSCs into mature neurons. Specifically, higher concentrations of MT (1 μM/10 μM) notably increased both the number and complexity of differentiated neurons (Fig. 2B, C). Immunostaining for neural markers (NFL, NeuN) at 21 days post-differentiation confirmed that MT significantly improves the neuronal differentiation efficiency (Fig. 2D, E). Furthermore, quantitative analysis of neuronal aggregations revealed a significant increase with MT treatment, indicating enhanced neuronal plasticity and functionality (Fig. 2F). These findings indicate that MT can promote the maturation and functional capabilities of differentiated neurons.

The above results indicate that MT significantly enhances the differentiation potential of hiPSCs into NSCs and neurons, as well as promotes the expression of midbrain neuronal markers. To further elucidate the effect of MT on the differentiation of hiPSCs into midbrain DA neurons, we employed a small molecule-induced three-step method to differentiate NSCs into DA neurons, incorporating with varying MT concentrations (Fig. 2G). The results demonstrated that higher concentrations of MT significantly augment the differentiation potential of DA neurons (Fig. 2H, I). Immunofluorescence analysis of DA neuronal markers Tyrosine Hydroxylase (TH), homeobox transcription factors Engrailed1 (EN1), and terminal neuronal markers Neurofilament light (NFL), neuronal nuclear protein‌ (NeuN) further confirmed that MT enhances the efficiency of NSC differentiation into DA neurons (Fig. 2J–L). Statistical analysis of neuronal aggregations of differentiated neurons also revealed that MT promotes the plasticity and functional maturity of differentiated DA neurons (Fig. 2M).

### MT promoted the neural differentiation potential of hiPSCs by regulating the MT receptor MT1/2

**Fig. 3:**
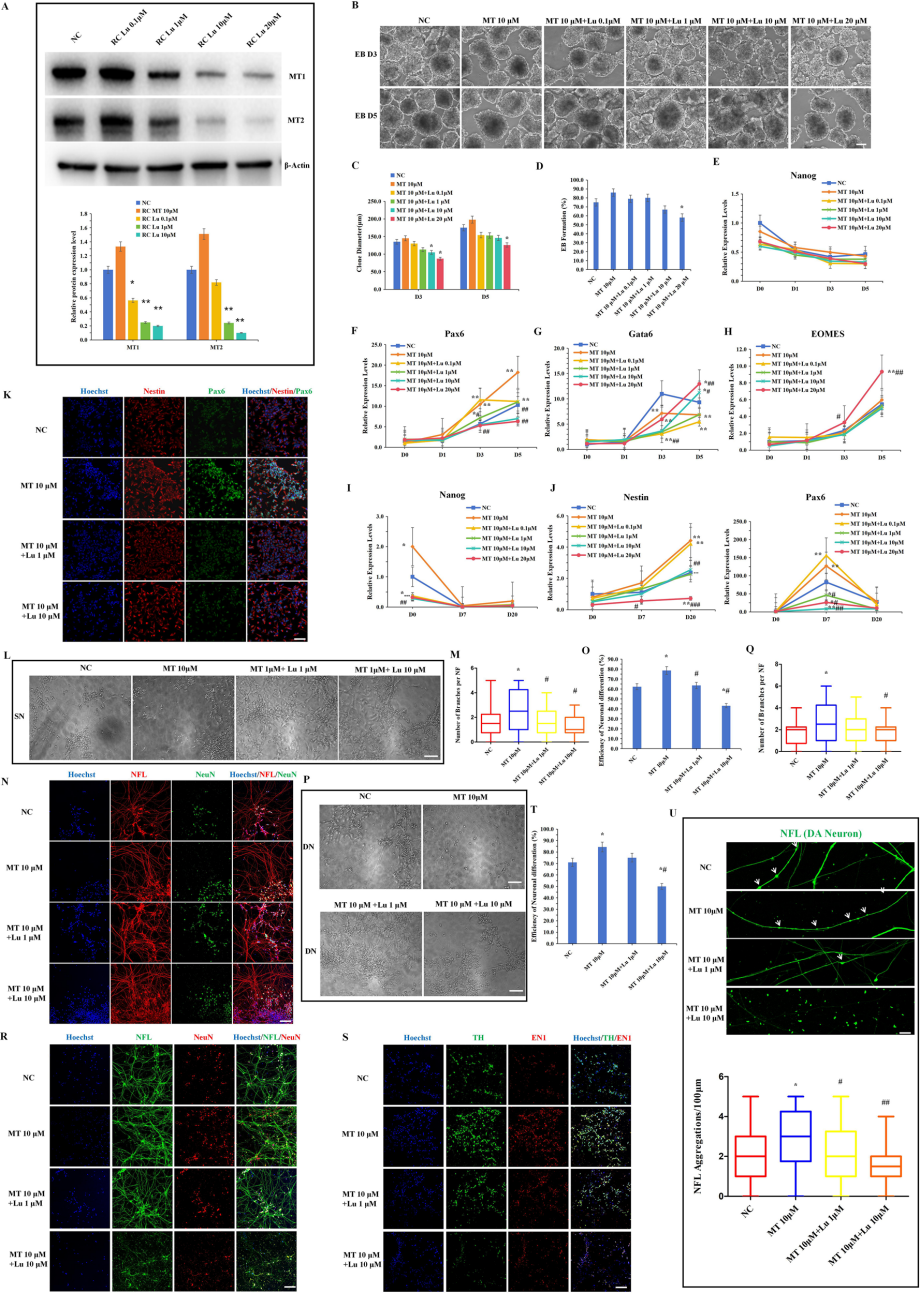
MT promoted the neural differentiation potential of hiPSCs by regulating MT receptor MT1/2. **A** Expression of MT1(12 KDa) and MT2(40 KDa) was determined by western blot after 7 days of Luzindole treatment in hiPSCs. The comparison between the target protein and the reference protein β-Actin(42KDa) revealed significant results. **B** The morphology of EB differentiated on days 3 and 5 after the combined treatment of Luzindole with MT. Scale bars, 50 μm. **C**, **D** The statistics of EB clone diameter and formation efficiency on day 5. **E-H** Expression of pluripotency marker (Nanog) and three germ layer markers (Pax6, Gata6, EOMES) was determined by qRT-PCR. **I**, **J** Expression of pluripotency marker (Nanog) and NSC markers (Pax6, Nestin) was determined by qRT-PCR. **K** Immunofluorescence staining of NSC markers (Pax6, Nestin). Scale bars, 50 μm. (**L**) The morphology of differentiated neurons with varying concentrations of Luzindole on 10 μM MT. Scale bars, 20 μm. **M** The statistics of number of branches per NF in spontaneously differentiated neurons. **N**, **O** Immunofluorescence staining of neuron markers (NFL, NeuN) and the differentiation efficiency statistics. Scale bars, 50 μm. **P** The morphology of differentiated DA neurons with varying concentrations of Luzindole on 10 μM MT. Scale bars, 20 μm. **Q** The statistics of number of branches per NF in directed differentiated DA neurons. **R**–**T** Immunofluorescence staining of DA neuron markers (TH, EN1, NFL, NeuN) and the differentiation efficiency statistics. Scale bars, 50 μm. **U** Immunofluorescence staining and quantitative analysis of DA neuronal aggregations. White arrows, neuronal aggregation. Scale bars, 10 μm.*P < 0.05, **P < 0.01, ***P < 0.001 *vs*. NC. #*P* < 0.05, ##*P* < 0.01 *vs*. MT 10 μM.

Numerous studies have reported that MT receptors MT1 and MT2 can prevent the apoptosis of brain neurons in neurodegenerative diseases by regulating metabolic signals [12]. However, other studies have suggested that MT can independently promote the differentiation of stem cells into functional cells, regardless of MT1 and MT2 [31]. To investigate whether MT promotes neuronal differentiation potential by regulating MT1 and MT2 receptors, we applied Luzindole, a potent MT receptor inhibitor, to suppress MT receptor protein expression. After seven days of Luzindole treatment, MT receptor protein expression significantly decreased in vitro (Fig. 3A). To further investigate whether MT’s promotion of hiPSCs neuronal differentiation depends on MT receptor, we conducted random differentiation of hiPSCs with varying concentrations of Luzindole added on top of 10 μM MT treatment to assess differences in germ layer differentiation potential among different treatment groups. The results indicated that higher concentrations of Luzindole (10 μM/20 μM) significantly attenuated MT’s promotion of EB random differentiation efficiency (Fig. 3B–D). Analysis of pluripotency and three germ layer marker expression revealed that Luzindole significantly suppressed MT’s effect on hiPSCs differentiation into neuroectoderm (Figs. 3E, F, S2A,B) and restored its inhibitory effect on endodermal differentiation (Figs. 3G, S2C). However, Luzindole did not significantly affect mesodermal differentiation, suggesting that MT’s influence on this lineage is independent of MT receptors (Figs. 3H, S2D). Moreover, to further investigate whether MT relies on MT1/2 receptors to promote NSC differentiation potential, we examined the combined effect of Luzindole with MT on hiPSCs-derived NSCs following the aforementioned differentiation procedures. The results demonstrated that Luzindole significantly inhibited MT’s enhancement of NSC differentiation potential (Figs. 3I–K, S2E-H). Additionally, Luzindole attenuated MT’s promotion of midbrain neuronal marker Nurr1 expression to some extent (Fig. S2F, J), indicating MT’s dependency on MT1/2 for enhancing midbrain lineage neuronal differentiation potential. Flow cytometry analysis further confirmed that Luzindole affected the expression of NSC markers (Pax6/Nestin/SOX1/SOX2) and midbrain neuronal marker Nuclear receptor related 1 (Nurr1) (Fig. S2H). Notably, CCK8 assay and Ki67 flow cytometry results indicated that Luzindole did not affect NSC self-renewal potential (Fig. S2H, I), thus ensuring that Luzindole’s impact on hiPSC neural differentiation potential was not due to alterations in cell proliferation signals.

**Fig. 4:**
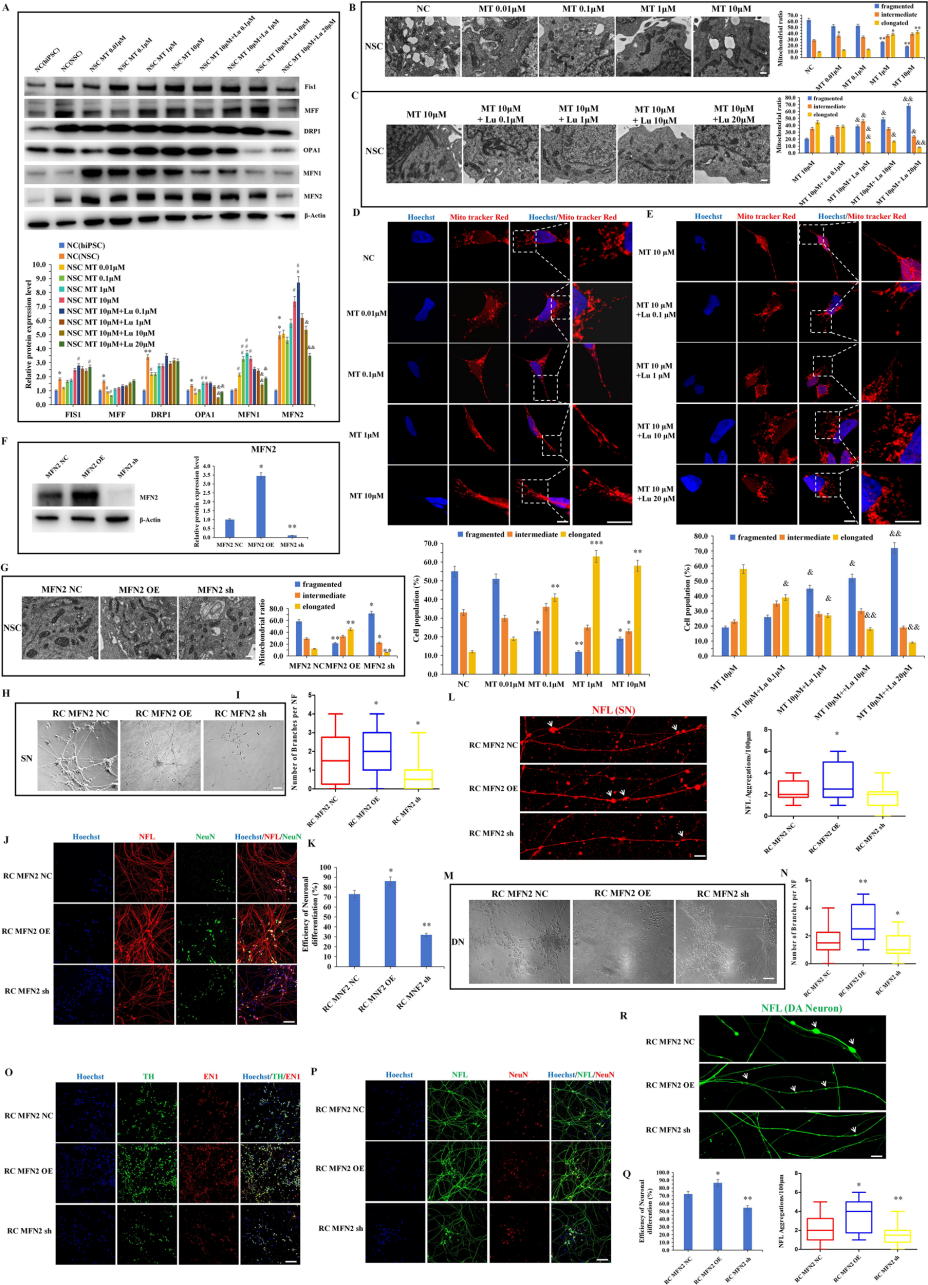
MT promotes mitofusin2 (MFN2)-mediated neurodifferentiation potential of hiPSCs. **A** Expression of mitochondrial fusion and fission-related proteins MFN1 (86KDa), MFN2 (88KDa), Fis1 (17KDa), DRP1 (1:1000) (80KDa), OPA1 (1:1000)(80-100KDa) and MFF (28-35KDa) was determined by western blot after 7 days of MT and Luzindole treatment in NSCs. β-Actin(42KDa) as the control group. **B**, **C** Transmission electron microscopy is used for statistical analysis of the transition of mitochondrial morphology after 7 days of MT and Luzindole treatment in NSCs. Mitochondria with a length less than 0.5 micrometers are defined as fragmented, those longer than 1 micrometer are elongated, and those in the middle are intermediate. Scale bars, 1 μm. **D**, **E** MitoTracker Red staining corroborated MT’s role in mitochondrial morphological transformation (fragmented, intermediate, elongated) through MT1/2 modulation. Scale bars (Normal), 20 μm; Scale bars (Amplified), 10 μm. **F** Expression of MFN2(88KDa) at the protein level was verified by Western blot. β-Actin(42KDa) as the control group. (**G**) Transmission electron microscopy analyzes the transition of mitochondrial morphology of MFN2 overexpressing and knockdown-hiPSCs. Scale bars, 1 μm. **H** The morphology of differentiated neurons derived from MFN2 overexpressing and knockdown-hiPSCs. Scale bars, 20 μm. **I** The statistics of number of branches per NF in the differentiated neurons. **J**, **K** Immunofluorescence staining of neuron markers (NFL, NeuN) and the differentiation efficiency statistics. Scale bars, 50 μm. **L** Immunofluorescence staining and quantitative analysis of neuronal aggregations. White arrows, neuronal aggregation. Scale bars, 10 μm. **M** The morphology of differentiated DA neurons derived from MFN2 overexpressing and knockdown-hiPSCs. Scale bars, 20 μm. **N** The statistics of number of branches per NF in directed differentiated DA neurons. **O**–**Q** Immunofluorescence staining of DA neuron markers (TH, EN1, NFL, NeuN) and the differentiation efficiency statistics. The efficiency of neuronal differentiation is demonstrated by the positive proportion of neuronal markers (such as the ratio of cells stained positively for EN1 in the cell bodies to the original inoculated cells. Scale bars, 50 μm. **R** Immunofluorescence staining and quantitative analysis of DA neuronal aggregations. White arrows, neuronal aggregation. Scale bars, 10 μm. *P < 0.05, **P < 0.01, ***P < 0.001 *vs*. NC (hiPSC)/RC MFN2 NC. #*P* < 0.05, ##*P* < 0.01 *vs*. NC (NSC). &*P* < 0.05, & &*P* < 0.01 *vs*. NSC MT 10 μM.

To elucidate whether MT promotes the differentiation potential of mature neurons through MT1/2 receptors. Initially, spontaneous differentiation of neurons revealed that 10 μM Luzindole markedly attenuated MT’s effects on neuronal differentiation potential, including efficiency and plasticity (Fig. 3L–O). Subsequently, we focused on targeted differentiation into DA neurons and assessed the effect of Luzindole on MT-mediated regulation of mature DA neuronal differentiation. Morphological differentiation and immunofluorescence analyses of DA neuron markers (TH, EN1, NFL, NeuN) indicated that 10 μM Luzindole significantly hindered MT’s promotion of DA neuron differentiation potential (Fig. 3P–T). Statistical analysis of neural filament aggregations in differentiated DA neurons further demonstrated that Luzindole notably suppressed MT-enhanced formation of neuronal aggregates (Fig. 3U).

### MT promotes mitofusin2 (MFN2)-mediated neurodifferentiation potential of hiPSCs

It has been reported that MT acts as a mitochondrial targeting hormone [31, 32] and may mitigate neurotraumatic diseases by influencing mitochondrial morphology [33–35]. Functional neural cells derived from pluripotent stem cells play a key role in the recovery of neurotraumatic diseases. However, the mechanism by which MT enhances functional neural differentiation of hiPSCs through modulation of mitochondrial dynamics remains unclear. Herein, we investigated the impact of MT and Luzindole (a target MT receptor inhibitor) on the expression of mitochondrial dynamics-related proteins in hiPSCs and differentiated NSCs. The results indicated that MT and Luzindole did not significantly affect the expression of mitochondrial dynamics-related proteins in hiPSCs (Fig. S3A). In contrast, NSCs differentiated from hiPSCs exhibited heightened mitochondrial kinetic activity and a more active mitochondrial fusion-fission process compared to hiPSCs after MT treatment, suggesting increased mitochondrial energy demand during NSCs differentiation. At the NSC level, increasing MT concentration significantly enhanced the expression of mitochondrial fusion proteins MFN1, MFN2, and Optic Atrophy 1 (OPA1) (Figs. S3B–D, 4A), with MFN2 showing the most pronounced effect. Immunofluorescence experiments confirmed MT’s effect on MFN2 expression (Fig. S3H, I). However, MT had no significant impact on the expression of mitochondrial fission-related proteins Dynamin-Related Protein 1 (DRP1), Mitochondrial Fission Factor (MFF) and Mitochondrial Fission 1 (Fis1) (Figs. S3E–G, Fig. 4A). Further analysis revealed that Luzindole markedly attenuated MT’s promotion of mitochondrial fusion protein expression in NSCs, a finding supported by immunofluorescence experiments (Figs. S3J-K, 4A). Result of transmission electron microscopy demonstrated that MT promoted the transition of mitochondrial morphology from fragmented to elongated rod-like structures (Fig. 4B), while higher concentrations of Luzindole (10 μM/20 μM) inhibited MT’s effect on mitochondrial fusion dynamics in NSCs (Fig. 4C). MitoTracker Red staining corroborated MT’s role in mitochondrial morphological transformation through MT receptor modulation (Fig. 4D, E).

Our previous results showed the essential role of mitochondrial dynamics in maintaining pluripotency and facilitating neural differentiation and development in pluripotent stem cells [36]. Specifically, the mitochondrial fusion dynamics protein MFN2 has been shown to enhance the NSC differentiation potential of hiPSCs [30]. We will further elucidate whether MFN2 can promote the directed differentiation of hiPSCs into mature neurons in the following study. First, we established hiPSCs cell lines (RC hiPSCs) with MFN2 overexpressing and knockdown separately (Fig. 4F). The impact of MFN2 overexpression and knockdown on mitochondrial morphology in hiPSCs was validated using transmission electron microscopy. Results revealed that MFN2 overexpression enhanced mitochondrial fusion dynamics, resulting in a higher proportion of mitochondria adopting elongated rod-like shapes. In contrast, MFN2 knockdown increased mitochondrial fission activity (Fig. 4G). Using the protocols of spontaneous neuronal differentiation and targeted DA neuronal differentiation, we assessed the effects of MFN2 manipulation on the mature neuronal differentiation potential of hiPSCs. Spontaneous neuronal differentiation assays demonstrated that MFN2 overexpression significantly promoted NSCs to neuron differentiation, enhancing the number of branches per neuronal filament, whereas MFN2 knockdown had the opposite effect (Fig. 4H, I). Immunofluorescence analysis of differentiated neurons revealed that MFN2 overexpression increased neuronal differentiation efficiency and the complexity of neuronal filament aggregation (Fig. 4J–L). Consistent with these findings, we applied the small molecule-mediated differentiation procedures to explore MFN2’s impact on the efficiency of NSC differentiation into targeted DA neurons. The results mirrored those of random neuronal differentiation, with MFN2 overexpression significantly enhancing NSC differentiation into DA neurons, including increasing the number of branches per neuronal filament (Fig. 4M, N). Furthermore, MFN2 overexpression augmented the efficiency of DA neuron differentiation and enhanced the aggregation and complexity of neuronal filaments in differentiated neurons (Fig. 4O–R). These results collectively demonstrate that MFN2 significantly improve both the differentiation efficiency and functionality of mature neurons, underscoring its role as a key target in MT-mediated regulation of neural differentiation.

### MT promotes MFN2-mediated WNT/β-catenin signal

**Fig. 5:**
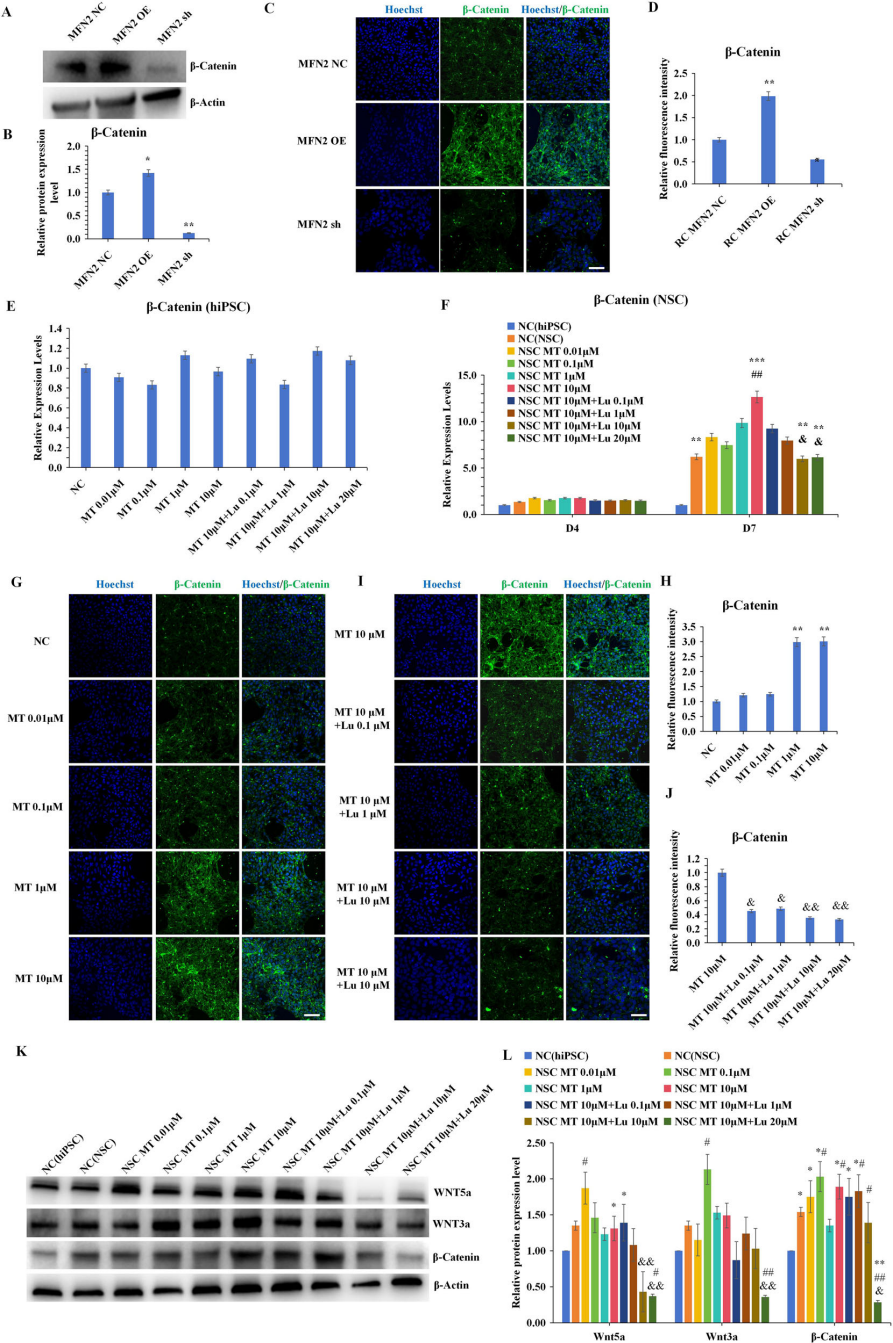
MT promotes MFN2-mediated WNT/β-catenin signal. **A**–**D** Western blot and immunofluorescence staining verified the expression of β-catenin(92KDa) in differentiated NSCs derived from MFN2 overexpressing and knockdown-hiPSCs. Scale bars, 50 μm. **E** Expression of β-catenin was determined by qRT-PCR after 7 days of MT and Luzindole treatment in hiPSCs. **F** Expression of β-catenin was determined in NSCs by qRT-PCR after 4 and 7 days of Luzindole treatment on 10 μM MT. **G**–**J** Immunofluorescence staining of β-catenin and the statistics of the relative fluorescence intensity after 7 days of MT and Luzindole treatment in NSCs. Scale bars, 50 μm. **K**, **L** Western blot verified the expression of WNT/β-catenin signaling pathway-related proteins WNT3a(40KDa), WNT5a(43KDa), and β-catenin(92KDa) after 7 days of MT and Luzindole treatment in NSCs. β-Actin(42KDa) as the control group. *P < 0.05, **P < 0.01 *vs*. MFN2 NC/NC (hiPSC). #*P* < 0.05, ##*P* < 0.01 *vs*. NC (NSC). &*P* < 0.05, & &*P* < 0.01 *vs*. NSC MT 10 μM.

Numerous studies have emphasized β-catenin’s role in maintaining mitochondrial homeostasis in metabolic diseases and mitigating mitochondrial toxicity in neurodegenerative disorders through activation of the WNT signaling pathway [29]. Additionally, MT has been reported to exert neuroprotective effects by activating β-catenin [28]. However, it remains unexplored whether MT influences neuronal differentiation efficiency by modulating MFN2-mediated WNT/β-catenin signaling at the hiPSC level and during neuronal differentiation. Initially, we confirmed that MFN2 overexpression significantly inscreased β-catenin expression in differentiated NSCs at the protein level (Fig. 5A, B), corroborated finding further supported by immunofluorescence results (Fig. 5C, D). However, we observed that MT and Luzindole did not affect β-catenin expression in hiPSCs significantly, confirming that MT doesn’t regulate β-catenin expression (Fig. 5E). This indicates that MT likely influences neural differentiation downstream or at the NSC level rather than through direct regulation of β-catenin expression in hiPSCs, suggesting its role in neural cell differentiation and NSC function modulation. To further test this hypothesis, we evaluated the impact of MT on β-catenin expression at the NSC level. Results showed that increasing concentrations of MT significantly enhanced β-catenin expression in NSCs, particularly in the later stages of differentiation (Fig. 5F–J). The addition of Luzindole effectively inhibited MT-induced β-catenin expression in NSCs (Fig. 5F–J), indicating that MT can indeed regulate β-catenin expression and is dependent on MT receptors. Furthermore, we investigated the effects of MT on WNT/β-catenin signaling pathway-related proteins at the NSC level. Increasing MT concentrations correlated with a general increase in the expression of WNT/β-catenin signaling pathway-related proteins (WNT3a, WNT5a, and β-catenin) in NSCs. Luzindole attenuated the effects of MT on the expression of these proteins (Fig. 5K, L). These findings collectively suggest that MT regulates neuronal differentiation efficiency, at least in part, through modulation of the MFN2-mediated WNT/β-catenin signaling pathway in NSCs.

### Dopaminergic neuroprogenitors (DAPCs) derived from MT-hiPSCs improved nerve regeneration and motor function in PD mice

**Fig. 6:**
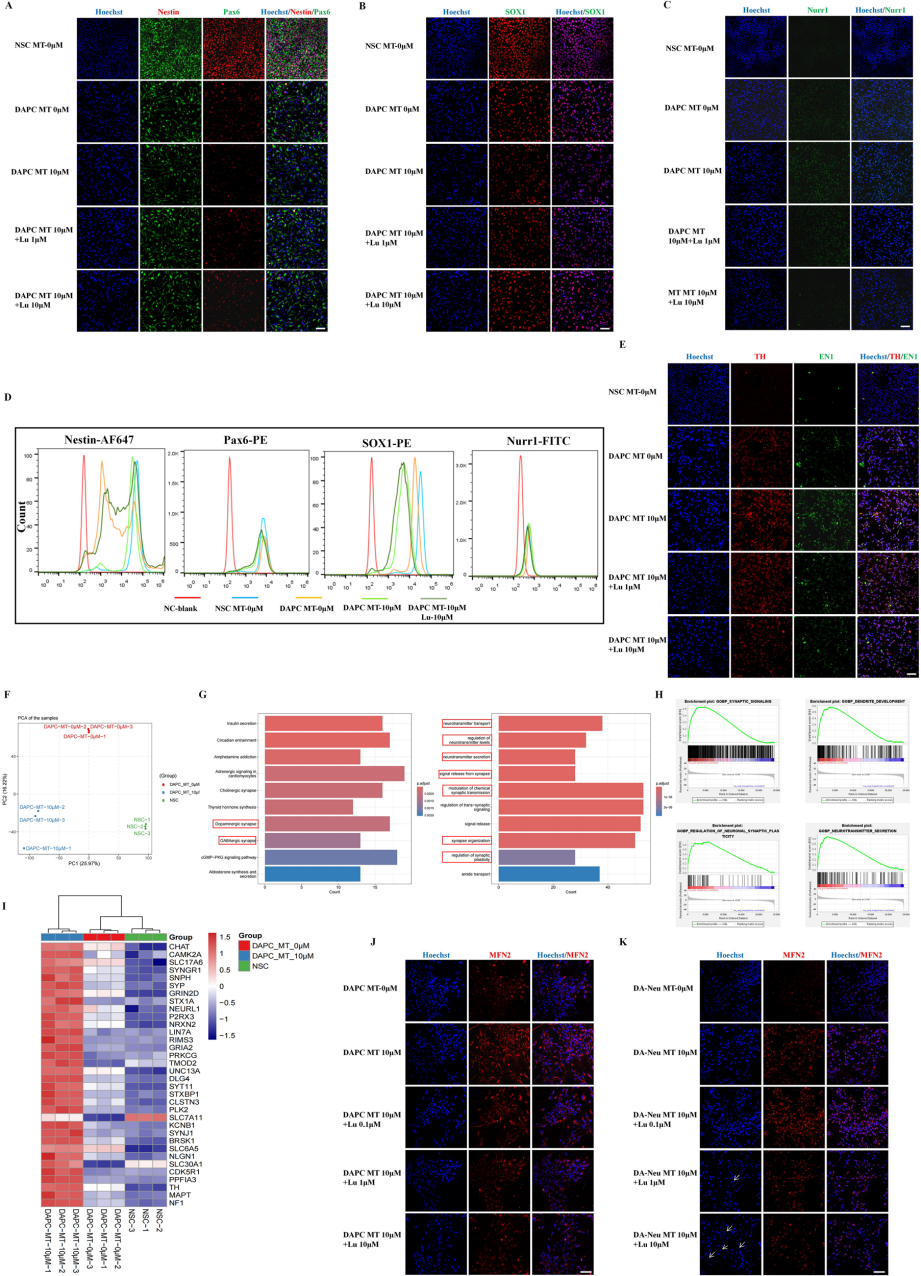
MT promoted the DAPCs differentiation potential of hiPSCs by regulating MT receptor MT1/2. **A**, **B** Immunofluorescence staining of NSC markers (Nestin, Pax6, SOX1) in NSCs and differentiated DAPCs. Scale bars, 50 μm. **C** Immunofluorescence staining of the midbrain neural marker Nurr1 in NSCs and differentiated DAPCs. Scale bars, 50 μm. **D** Flow cytometry analysis of NSC markers (Nestin, Pax6, SOX1) and the midbrain neural marker Nurr1. **E** Immunofluorescence staining of DA neuron markers (TH, EN1) in NSCs and differentiated DAPCs. Scale bars, 50 μm. **F** PCA analysis from RNA-Seq among NSCs and DAPCs before and after 10 μM MT treatment. **G**, **H** Gene ontology (GO) and Gene Set Enrichment Analysis (GSEA) from RNA-seq between DAPCs MT 0 μM and DAPCs MT 10 μM. **I** Heat map view of selected gene sets associated with neurons and synapses from RNA Seq of DAPCs MT 0 μM and DAPCs MT 10 μM. (**J**) Immunofluorescence staining of MFN2 in the differentiated DAPCs. Scale bars, 50 μm. **K** Immunofluorescence staining of MFN2 in the differentiated DA neurons. Scale bars, 50 μm.

DAPCs are neural stem/progenitor cells that exhibit closer functional characteristics to DA neurons compared to NSCs. Similar to NSCs, they possess high self-renewal and differentiation potential. Consequently, we employed DAPCs derived from NSCs for PD cell therapy. Initially, hiPSCs-NSCs were directed to differentiate into DAPCs over 7-10 days using a midbrain neural differentiation scheme. Immunofluorescence results indicated reduced expression of NSC markers in the differentiated DAPCs, with MT enhancing the differentiation process of DAPCs. Luzindole further attenuated the MT’s effect on promoting DAPC differentiation (Fig. 6A, B). To confirm the identity of DAPCs, we assessed the expression of the midbrain neural marker Nurr1. Our differentiation protocol successfully differentiated NSCs into Nurr1-positive DAPCs, with MT enhancing Nurr1 expression, while Luzindole exhibited a certain inhibitory effect (Fig. 6C). Furthermore, we assessed the effects of MT on DAPC differentiation using flow cytometry (Fig. 6D). Surprisingly, the expression of DA neuronal markers (TH/EN1) significantly increased in MT-treated DAPCs, suggesting that MT enhances the efficiency of differentiation into DA neurons. Luzindole, however, inhibited the expression of DA neuronal markers in MT-treated DAPCs (Fig. 6E).

Further, we performed RNA-Seq (Fig. S4A), which confirmed that the optimized DAPCs-directed differentiation system can differentiate NSCs into DAPCs with functions related to neuron and synaptic development, and the gene expression of neurons and synapse-related pathways is significantly up-regulated (Fig. S4B–E). Meantime, MT can promote the synaptic plasticity of DA neurons and neurotransmitter secretion function of DAPCs (Fig. 6F–H, S4F–H), and the gene expression of the related signaling pathway, including TH, is significantly up-regulated (Fig. 6I). To detect the expression of MFN2 in MT-NSC differentiated DAPC, immunofluorescence results showed that MT significantly promoted the expression of MFN2 in DAPC, while Luzindole inhibited this effect of MT (Fig. 6J). Meantime, immunofluorescence results of further differentiation of DAPC into DA neurons showed that MT significantly promoted the expression of MFN2 in differentiated DA neurons, while Luzindole inhibited this effecct and led to nerve cell apoptosis to a certain extent (Fig. 6K).

**Fig. 7:**
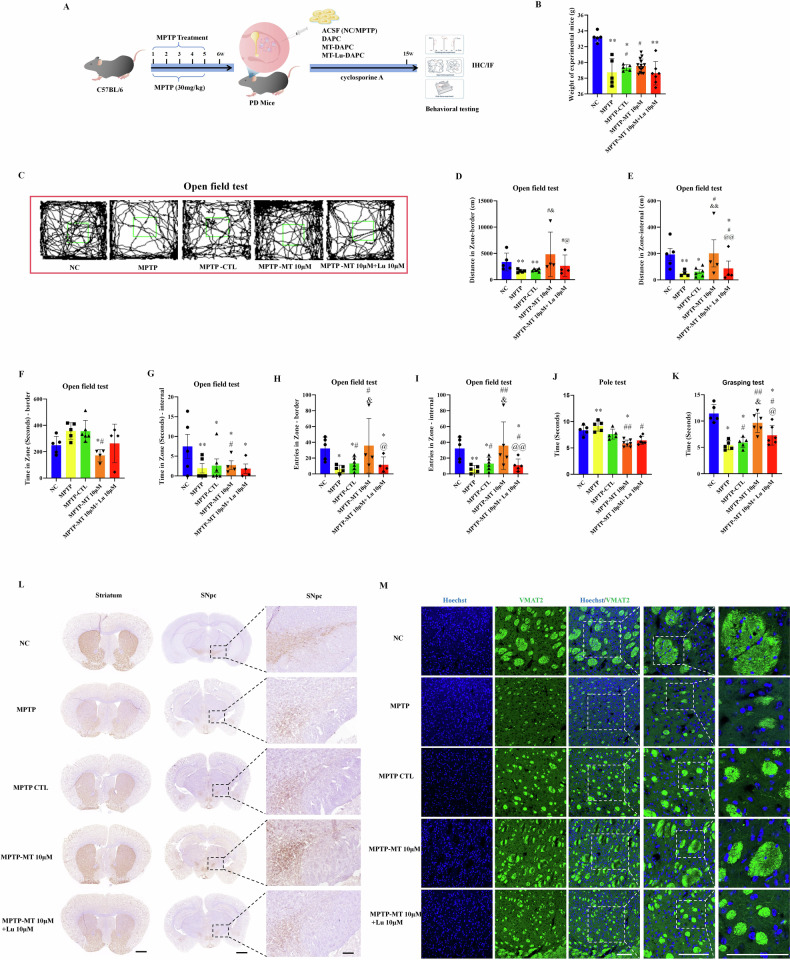
DAPCs derived from MT-hiPSCs improved nerve regeneration and motor function in PD mice. **A** Scheme of DAPC in situ treatment of PD mice. **B** Body weight statistics of PD mice after cell therapy. **C**–**I** Assessment of the distance of spontaneous motion in an open field. **J** Assessment of climbing time from top to bottom of the pole in the pole test. **K** Quantitative analysis of forelimb grasping time in the grasping test. **L** TH **i**mmunohistochemistry was examined in the striatum and SNpc sections of PD mice. Normal striatum and SNpc: scale bars, 1 mm. Amplified SNpc: scale bars, 100 μm. **M** VMAT2 immunofluorescence was examined in the striatum sections of PD mice. Scale bars, 20 μm. Body weight and behavioral tests were measured following a 2-month treatment period with cell injections. We used a double-blind method to group the animals and used the same amount of artificial cerebrospinal fluid as a strict control. *P < 0.05, **P < 0.01 *vs*. NC. #*P* < 0.05, ##*P* < 0.01 *vs*. MPTP. &*P* < 0.05, & &*P* < 0.01 *vs*. MPTP-CTL. @*P* < 0.05, @@*P* < 0.01 *vs*. MPTP-MT 10 μM.

We further investigated the therapeutic potential of MT-DAPC in promoting cerebral nerve regeneration and motor function recovery in PD mice, we designed an experimental scheme of DAPC in situ treatment of PD mice (Fig. 7A). First, we established a chronic PD mouse model by administering intraperitoneal injections of 1-methyl-4-phenyl-1,2,3,6-tetrahydropyridine (MPTP) (30 mg/kg) over 5 weeks. Then, mice exhibiting PD symptoms were selected for targeted striatal injection of DAPCs. To prevent immune rejection, cyclosporine A (30 mg/kg) was administered one week prior to cell injection. Following a 2-month treatment period with cell injections, behavioral tests, including the pole test, open field test, and grasping test, were conducted to assess motor function recovery in PD mice across different treatment groups. The results demonstrated significant improvement in weight and motor function in the DAPC-treated PD group. MT-DAPC treatment further enhanced motor function recovery compared to the DAPC group, while Luzindole attenuated the functional benefits of DAPC to some extent (Fig. 7B–K). Additionally, we evaluated cerebral nerve regeneration post-therapy through TH immunohistochemistry in the midbrain and striatum. The strongest expression of TH-positive neurons was observed in the MT-DAPC treatment group, indicative of MT1/2-dependent effects (Fig. 7L). Meantime, we also identified the promoting effect of MT on DAPC of striatal nerve regeneration through postsynaptic neuron marker Vesicular Monoamine Transporter 2 (VMAT2)-immunofluorescence, which is also dependent on MT1/2 (Fig. 7M). These findings underscore MT’s potential to enhance motor function recovery and midbrain DA nerve regeneration in PD treatment using DAPCs.

## Discussion

MT (N-acetyl-5-methoxytryptamine) is synthesized and secreted by the pineal gland, playing pivotal roles in various physiological processes such as circadian rhythm regulation, cell cycle control, autophagy, and neuroprotection [37, 38]. Notably, disrupted circadian rhythms are a hallmark of neurodegenerative diseases like PD, Alzheimer’s disease (AD), and Huntington’s disease (HD) [8, 9]. Alterations in circadian rhythms can significantly impact and exacerbate the aggregation of pro-neurodegenerative molecules [39]. Therefore, targeting the circadian rhythm system may offer new avenues for diagnosing and treating neurodegenerative diseases. A significant decrease in MT levels during aging is a characteristic feature of neurodegenerative diseases. The MT receptors MT1 (encoded by MTNR1A) and MT2 (encoded by MTNR1B) are crucial in regulating the downstream signaling pathway of MT. Research indicates that neuronal-specific overexpression of MT receptor MT1 reduces infarct size following ischemia/reperfusion, thereby enhancing nerve regeneration function [40]. In PD mouse models, decreased MT1 expression in microglial cells of the substantia nigra suggests a potential role of MT1 in PD pathogenesis [41]. Moreover, several studies have demonstrated that reductions in MT1 and MT2 levels in specific brain regions may contribute to the development of PD [12]. Previous studies have indicated that increased levels of MT1 and MT2 are associated with AD and ischemic neuronal injury, respectively, whereas decreased levels are observed in PD. Antagonists of MT1/MT2, such as Luzindole and the selective MT2 antagonist 4P-PDOT, are commonly used in research [42]. This study further explored how Luzindole at specific concentrations could inhibit MT’s promotion of hiPSCs’ differentiation into functional neurons. Additionally, it verified MT’s neuroprotective effect in the brain, mediated through MT1/2, in a PD mouse model. Therefore, enhancing MT levels to regulate DA content presents a promising avenue for clinically treating PD.

Research has demonstrated that MT induces the differentiation of NSCs into oligodendrocytes and neurons, a process dependent on increasing mitochondrial mass, respiration, membrane potential, and ATP synthesis in NSCs. In PD mouse models, combining NSC transplantation with pharmacological doses of MT effectively restored neuronal populations by enhancing mitochondrial activity [22]. This study explored the efficacy of MT-treated hiPSCs-derived DAPCs, which exhibited improved functionality and efficiency in regenerating PD midbrain nerves and highlights MT’s potential to increase the neuroprotective effects of DAPCs in vivo, suggesting its clinical utility in combination with neural progenitor cells from hiPSCs for PD treatment. Pharmacological doses of MT serve a dual role in NSC differentiation, enhancing oxidative phosphorylation (OXPHOS) and promoting the scavenging of reactive oxygen species (ROS) by boosting mitochondrial activity. This research contributes to our understanding of MT’s neuroprotective effects during neural differentiation and regeneration, providing a solid foundation for considering MT as a promising therapeutic target in PD treatment.

Several studies have demonstrated that MT enhances SVZ-NSC neurogenesis by upregulating mature neuronal markers like β-III-tubulin (Tuj-1) and TH, while concurrently downregulating the astrocyte marker glial fibrillary acidic protein (GFAP) [22]. Interestingly, experimental evidence suggests that mitochondria are a primary intracellular target of MT [43]. MT is proposed to regulate mitochondrial function by enhancing oxidative phosphorylation efficiency, ATP synthesis, and respiratory complex activity. However, the mechanisms through which MT influences mitochondrial dynamics and downstream signaling pathways affecting functional neuronal differentiation from pluripotent stem cells, as well as its impact on nerve regeneration in vivo, remain to be fully elucidated. The balance of mitochondrial dynamics is essential for maintaining cellular and physiological functional homeostasis. Dysregulation of proteins that control mitochondrial fusion and fission dynamics can contribute to various diseases, including neurodegenerative disorders [44]. Interestingly, mitochondrial dynamics exhibit an oscillatory pattern that aligns with the circadian secretion rhythm of MT [32], indicating a reciprocal relationship between MT and mitochondrial dynamics. MT is recognized as a mitochondrial-targeting hormone, characterized by its high concentrations within mitochondria and its ability to penetrate the mitochondrial membrane [45]. Studies have revealed that MT activates mitochondrial fusion and suppresses fission through the AMP-activated protein kinase (AMPK)-OPA1 axis, thereby mitigating myocardial and cerebral ischemia-reperfusion injury as well as vascular calcification [35]. Physiological levels of MT have been shown to enhance mitochondrial fusion during odontoblast differentiation, while depletion of OPA1, a key regulator of mitochondrial fusion, impairs MT-mediated differentiation and mitochondrial respiratory function [31]. Evidence also supports the involvement of mitochondrial dynamics in neurological disorders, where disruptions in mechanisms controlling mitochondrial morphology contribute to developmental defects in the brain. The mitochondrial fusion protein MFN2 is critical for neuronal maturation and synapse formation; conditional knockdown of MFN2 specific to the cerebellum leads to reduced cerebellar size and motor impairments. Collectively, these findings underscore the pivotal role of mitochondrial dynamics in brain neurodevelopment [46].

Biochemical and pathological anatomical studies of PD patients’ brains showed that impaired mitochondrial function is closely linked to neurodegeneration in PD. Experimental models involving loss of function in Parkin, PINK1, PARK7 (DJ-1), and Omi/HtrA2 genes have highlighted the essential role of mitochondrial dynamics in maintaining neuronal mitochondrial function in PD. MT pretreatment has been demonstrated to protect against the damaging effects of Prion virus PrPSc on mitochondrial function and dynamics, preserve synapses, and mitigate neuronal damage [47]. Therefore, understanding the protective signaling network involving mitochondria could reveal new therapeutic targets for PD through molecular modeling of mitochondria and pharmacological modulation of mitochondrial dynamics [48]. Herein, our study further elucidates that MT promotes mitochondrial fusion in NSCs via MT1/2 receptors and reduces mitochondrial fission to some extent. However, MT does not significantly influence mitochondrial dynamics at the hiPSCs level, suggesting that its effects on mitochondrial balance are more pronounced during neural differentiation of hiPSCs and NSCs. Importantly, our findings indicate that MT enhances NSC differentiation into functional DA neurons by upregulating MFN2 expression, underscoring the potential application of targeting the MT-MFN2 signaling axis for generating functional DA neurons in vitro, which holds significant promise for PD therapy.

WNT signaling plays an important role in embryonic development and regulates the maintenance, self-renewal, and differentiation of mammalian stem cells. The Studer group has proposed a protocol for deriving mDA neurons by activating WNT signaling in two steps, which enhances the expression of midbrain markers like EN1 while minimizing contamination from hindbrain and diencephalon lineages [18]. β-catenin, a key component of cadherin protein complexes, acts as a signal transducer in the WNT/β-catenin pathway. Lehwald et al. demonstrated that β-catenin regulates hepatic mitochondrial homeostasis and ATP production, indicating its role in mitochondrial function [28]. Activation of WNT/β-catenin signaling by WNT1 has been shown to protect against mitochondrial neurotoxicity induced by 6-OHDA in PD cell models, suggesting neuroprotective effects [49]. Studies have also linked β-catenin expression to PD pathology, where MPTP-induced neurotoxicity correlates with decreased β-catenin levels in a mouse model [50]. MT, known for its regulatory role across multiple organs, influences the WNT/β-catenin pathway [51]. Exogenous WNT1 has been found to protect SH-SY5Y cells from 6-OHDA-induced neurotoxicity by activating WNT/β-catenin signaling in PD processes [49]. Moreover, Gutierrez-Cuesta et al. observed that MT stimulates β-catenin expression in an aging mouse model, highlighting its role in regulating β-catenin levels [52]. In our study, we demonstrated that MT promotes activation of WNT/β-catenin signaling, evidenced by increased expression of WNT3a, WNT5a and β-catenin, aligning with previous findings on the regulatory mechanisms of WNT/β-catenin signaling in neural regulation. The results also showed that MT’s regulation of mitochondrial dynamics contributes to enhancing the functional neuronal differentiation potential of hiPSCs. These results highlight the interplay between mitochondrial function, WNT/β-catenin signaling, and neural differentiation, providing insights into potential therapeutic targets for PD treatment.

**Fig. 8:**
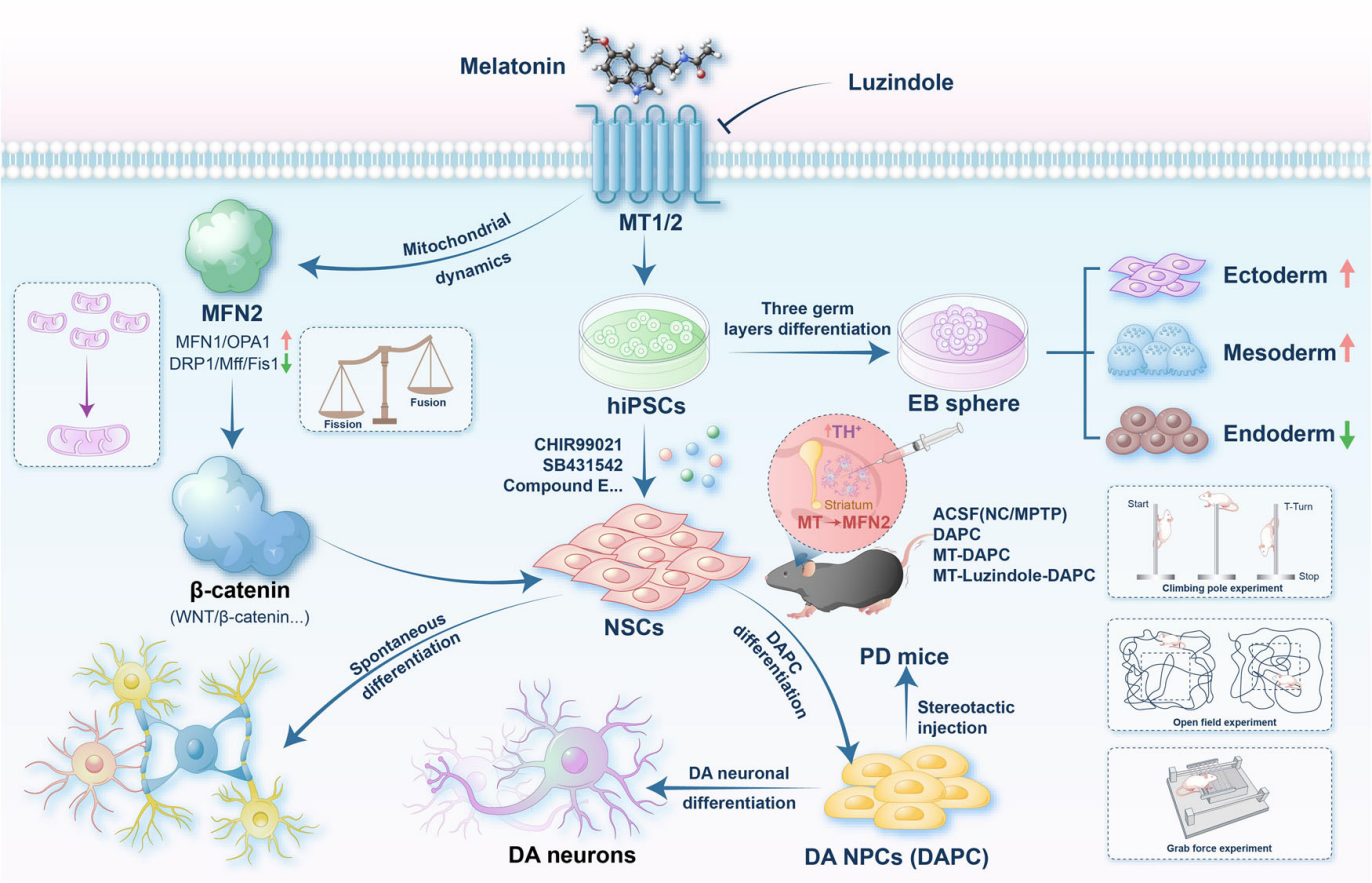
MT-targeted MT1/2 orchestrates mitochondrial fusion dynamics-mediated WNT/β-catenin signaling to promote DA neuronal differentiation of human iPS and nerve regeneration in PD mice. MT enhances the efficiency of directed functional DA neuron differentiation from hiPSCs by mediating MT1/2 receptors and subsequently promoting WNT/β-catenin signaling regulated by mitochondrial fusion dynamics. Moreover, MT significantly promoted nerve regeneration and improved motor function in PD mice models.

This study investigates the efficacy and functional enhancement of directed DA neuronal differentiation from hiPSCs through MT supplementation in vitro. Future research could further assess the functional maturity of differentiated DA neurons and validate neural regeneration in PD mouse models in vivo. The mitochondrial metabolic mechanisms and metabolic reprogramming induced by MT in regulating neural differentiation and regeneration of stem cells, both in vitro and in vivo, are essential areas for further exploration. Additionally, the survival rate of the transplanted cells and their distribution within the brain need to be further precisely identified and confirmed. And characterizing the key features of differentiated DAPC cells in vitro and evaluating their immune response effects in PD treatment in vivo (with the inclusion of cyclophosphamide in this study) requires further validation. In summary, our findings elucidate that MT enhances the efficiency of directed functional DA neuron differentiation from hiPSCs by mediating MT1/2 receptors and subsequently promoting WNT/β-catenin signaling regulated by mitochondrial fusion dynamics. Moreover, we confirm MT’s significant impact on promoting nerve regeneration and improving motor function in PD mice models (Fig. 8), underscoring the clinical relevance of targeting the MT-MFN2 axis for PD therapy and hiPSC-mediated stem cell combination therapies.

## Materials and methods

### Cell culture

The research-grade hiPSC cell line (REF: RC01001-A) (Ethical code number: ASSCR-YXLL-2019-03) was obtained by reprogramming the peripheral blood mononuclear cells of a 36-year-old healthy male through a non-integrative method, which was tested negative for mycoplasma., and is suitable for stem cell research and application in scientific research. HiPSCs were cultured in mTesR1 medium on Matrigel-coated plates at passages 20-30. Briefly, the hiPSCs were maintained by daily medium replacement and passaged every 3-4 days using 0.5 mM EDTA for enzymatic dissociation into small monolayer colonies. The passage was conducted at a 1:10 ratio using a 5 mL pipette. HiPSC-derived NSCs were cultured in DMEM/F12 supplemented with 1× N2, 1× B27, 1% GlutaMAX, 1% penicillin/streptomycin, 1% nonessential amino acids, 0.1 mM β-mercaptoethanol, 10 μg/mL BSA, 10 ng/mL hLIF, 4 μM CHIR99021, and 3 μM SB431542. The NSCs were passaged using Accutase at a 1:6 dilution. NSCs-derived DA neural progenitor cells (DAPCs) were cultured in DMEM/F12, 1×N2, 1×B27, 300 ng/mL cAMP, 0.2 µM VC, 100 nM LDN-193189, 600 ng/mL +/−Noggin, 100 ng/mL SHH, and 100 ng/mL FGF8b. All cells were maintained in a CO_2_ incubator at 37°C.

### Embryoid Body (EB) formation

EB formation assay was conducted following an optimized protocol. HiPSCs in the logarithmic growth phase were washed twice with DPBS and then dissociated into large clusters using 1 mg/mL dispase II for 15 minutes at 37 °C. The cells were mechanically dissociated using a 200 µL yellow pipette tip, collected, and centrifuged at low speed (300 rpm) for 5 minutes. After two additional washes with DMEM/F12 and centrifugation, the supernatant was carefully removed, and the cell clusters were resuspended in an EB formation medium containing Knockout DMEM, 20% KOSR, 1% GlutaMAX, 1% nonessential amino acids (NEAA), 8 ng/mL bFGF, and 10 µM Y27632, and the cell suspension was inoculated into low-adherent cell culture dishes. Y27632 was included initially to ensure high survival of the EB clusters and was removed after 24 hours. Lastly, the EB medium was replaced every two days by low-speed centrifugation (300 rpm for 3 minutes), and EBs were collected from days 1 to 5 to assess the expression of three blastodermal markers.

### Quantitative RT-PCR (qRT-PCR) Analysis

qRT-PCR was performed as previously described [36]. Total RNA was extracted using Trizol (AG RNAex Pro RNA extraction reagent, AG21101), followed by reverse transcription to cDNA using the Evo M-MLV RT Kit (AG11728), following the manufacturer’s protocol. qRT-PCR was conducted using the SYBR Green Premix Pro Taq HS qPCR Kit on the LightCycler^®^ 480 II System (Roche), following the kit instructions. The reference gene used in this experiment was β-actin. The relative quantification of the target gene was performed using the ΔΔCt method. The experiment was replicated biologically three times. Then, the GraphPad software was used for plotting and analysis. The data passed the normality test (Gaussian distribution) and homogeneity of variance test. The t test was used to compare the two groups (the target gene and the reference gene) and obtain significant results. The details of the primers used are provided in the Supplementary Table 1.

### Immunofluorescence Staining

For this experiment, the cell samples were fixed in 4% paraformaldehyde (PFA) for 15 minutes, permeabilized with 0.125% Triton X-100 for 20 minutes, and blocked with DPBS containing 5% BSA for 1 hour. They were then incubated overnight at 4°C with primary antibodies (Supplemental Table 2) against Nanog (1:200)(RRID:AB_2536677), OCT4 (1:400)(RRID:AB_628051), SSEA3 (1:200)(RRID:AB_2536686), TRA-1-81 (1:400)(RRID:AB_2536706), Nestin (1:100) (RRID:AB_2799037), PAX6 (1:200)(RRID:AB_2797599), SOX1 (1:400)(RRID:AB_2809724), SOX2 (1:400) (RRID:AB_2536667), Nurr1 (1:100)(RRID:AB_2298676), NFL (1:100) (RRID:AB_632015), NeuN (1:100)(RRID:AB_2651140), TH (1:100)(RRID:AB_628422), EN1 (1:100)(RRID:AB_2246492), MFN2 (1:100)(RRID:AB_2716838), β-catenin (1:100) (RRID:AB_11127855) and VMAT2 (1:100)(RRID:AB_11154073). Then, the cells were washed and incubated with the fluorescent secondary antibodies (1:200) for 1 hour at room temperature (RT). Nuclei were stained with Hoechst 33342 (10 mg/mL) for 10 minutes at RT in the dark. Immunofluorescence images were taken immediately using a laser scanning confocal microscope (Zeiss LSM 780), and fluorescence intensity data were analyzed using ZEN microscope imaging software.

### Western blotting

Equal amounts of cell lysates were separated by SDS-PAGE, transferred to a nitrocellulose (NC) membrane, and blocked with 5% milk. Monoclonal primary antibodies (Supplemental Table 4) against β-actin (1:1000)(RRID:AB_2536844, 42KDa), MFN1 (1:1000)(RRID:AB_2809773, 86KDa), MFN2 (1:1000)(RRID:AB_2716838, 88KDa), Fis1 (1:1000)(RRID:AB_11152577, 17KDa), DRP1 (1:1000) (RRID:AB_2897963, 80KDa), OPA1 (1:1000)(RRID:AB_2644984, 80-100KDa), MFF (1:1000) (RRID:AB_2639010, 28-35KDa), β-catenin (1:1000)(RRID:AB_11127855, 92KDa), WNT3a (1:1000)(RRID:AB_2851726, 40KDa), WNT5a (1:1000)(RRID:AB_10986099, 43KDa, 58KDa), MT1 (1:1000)(RRID:AB_2719477, 12KDa), and MT2 (1:1000)(RRID:AB_2851534, 40KDa) were used at appropriate dilutions. HRP-conjugated anti-rabbit or anti-mouse secondary antibodies (1:50,000) were incubated for 1 hour at RT, and protein bands were visualized using the Western ECL Substrate (Bio-Rad).

### NSC differentiation

HiPSCs at 30% confluence were dissociated into single cells using Accutase and cultured in NSC differentiation media supplemented with 3 µM SB431542, 4 µM CHIR99021, and 0.1 µM Compound E. The differentiation media consisted of DMEM/F12 (1:1), 1×N2, 1×B27, 1% GlutaMAX, 10 µg/mL BSA, and 10 ng/mL hLIF, for 7 days. After induction, the cells were subcultured at a 1:3 ratio using Accutase for six passages on Matrigel-coated plates, followed by continued differentiation in NSC induction media supplemented with 3 µM SB431542 and 4 µM CHIR99021. Subsequently, NSCs were passaged at a 1:6 ratio every 3 days. NSCs from the two-step induction process were collected at various time points for identification of NSC markers (Nestin, Pax6, SOX1, SOX2, Nurr1) using immunofluorescence staining and flow cytometry analysis. Initially, 10 µM ROCK inhibitor Y27632 was added to enhance cell survival during the early passages, but was unnecessary in subsequent passages.

### Neuronal differentiation

Neuronal differentiation was initiated on Matrigel- and poly-l-ornithine-coated surfaces. Single-cell spontaneous differentiation was induced in a neuronal induction medium composed of DMEM/F12, 1×N2, 1×B27, 300 ng/mL cAMP, and 0.2 µM VC. Approximately 1000 differentiated NSCs were plated in a 6-well plate in a neuronal induction medium supplemented with 4 μM CHIR99021, and 3 μM SB431542 for three days. Subsequently, the medium was changed to include 4 μM CHIR99021, 3 μM SB431542, and 100 nM LDN-193189. After three days, the medium was changed to a differentiation medium containing 10 ng/mL BDNF and 10 ng/mL GDNF for 11-15 days. For DA neuronal differentiation, NSCs were first treated with a differentiation medium containing 3 μM SB431542 and 4 μM CHIR99021 for 3 day. Subsequently, the medium was changed to include 3 μM SB431542 and 100 nM LDN-193189 for 1 days. Next, the medium was changed to LDN-193189 (100 nM), +/−Noggin (600 ng/mL), SHH (100 ng/mL), and FGF8b (100 ng/mL) for 10 days. At this stage, the cells were induced to DAPCs. Lastly, DAPCs were treated with differentiation medium containing wnt-1 (100 ng/mL), BDNF (10 ng/mL), GDNF (10 ng/mL), IGF1 (10 ng/mL), TGF-β3 (2 ng/mL), DAPT (1 μM), and db-cAMP (1 mM) for an additional 14-21 days to promote further differentiation. The specific markers of differentiated neurons were assessed using immunofluorescence staining.

### Flow Cytometry

HiPSC-derived NSCs and DAPCs were stained with surface marker antibodies for flow cytometry analysis. Cells were dissociated with Accutase for 3 minutes at 37°C, then approximately 1×10^6^ cells were used per sample. The cells were incubated on ice for 1 hour, fixed with a specialized fixing solution for 15 minutes at RT, and washed twice with DPBS. For samples with conjugated antibodies (Supplemental Table 3), the cells were incubated with the antibodies for 30 minutes at 4°C, washed with 10% FBS-HBSS, and analyzed using FACS. Background staining was controlled using appropriate controls. Each sample recorded 30,000 events, and the analysis was repeated at least twice per cell line.

### PD mice model construction and stem cell stereotaxic injection

Male C57BL/6 mice aged 6 weeks were divided into five groups: Artificial cerebrospinal fluid (ACSF), MPTP, MPTP + hiPSCs-DAPCs, MPTP + hiPSCs-MT-DAPCs, and MPTP + hiPSCs-MT (Luzindole)-DAPCs, with each group comprising 10 mice. To establish a chronic PD mouse model, the mice were intraperitoneally injected with 30 mg/kg MPTP twice a week for 5 weeks. Only mice exhibiting less than 15 seconds on the suspension rod were selected for the subsequent cell transplantation experiments. Approximately one week after model induction, stereotactic injection of 2 × 10^5^ cells (100,000 cells/μL) in 2 μL ACSF with 10 μM Y27632 was performed using a microinjector into the right striatum ([AP] = +0.6 mm, [L] = -1.8 mm, [V] = -3.2 mm). The injection rate was 0.5 μL/min, and the needle remained in place for an additional 5 minutes before slow retraction. To prevent graft rejection, all animals received immunosuppression with cyclosporine A injections (30 mg/kg) starting one day before surgery, followed by injections every other day at 10 mg/kg. Two months post-injection, PD mice were euthanized, and brain tissue was collected for immunohistochemical staining of neuronal markers. Concurrently, behavioral assessments were conducted using a pole test and an open field test to evaluate exercise ability, as well as a grasping test to assess movement coordination. All animal experimental protocols were approved by the Ethical Committee of Peking University Shenzhen Hospital.

### Statistical Analysis

The data are presented as means ± standard deviation (SD). Statistical analysis was performed using the Student’s *t*-test and ANOVA analysis with GraphPad Prism 9. A p-value ≤ 0.05 was considered statistically significant, denoted by *p < 0.05, **p < 0.01, ***p < 0.001, #*p* < 0.05, ##*p* < 0.01, ###*p* < 0.001. &*p* < 0.05, & &*p* < 0.01, & & &*p* < 0.001, @*p* < 0.05, @@*p* < 0.01, All data are representative of at least 3 independent biological replicates to match the statistical standards of different experiments. Samples in the experiment were randomly selected for measurement and statistics, and the samples that deviated by more than 10 times from the median were excluded. The variance is similar between the groups that are being statistically compared.

## Supplementary information

Supplementary Figure Legends

Supplementary Figure 1

Supplementary Figure 2

Supplementary Figure 3

Supplementary Figure 4

Western blot_1

Supplementary Table 1

Supplementary Table 2

Supplementary Table 3

Supplementary Table 4

## Data Availability

Further information and requests for resources and regents should be directed to and will be fulfilled by the Lead Contact, Peng Cui (pengcui@pkuszh.com).

## Code availability

All the datasets uploaded could be accessed on the URL: https://www.ncbi.nlm.nih.gov/geo/query/acc.cgi?acc=GSE295508. Accession “GSE295508” is currently private and the secure code is “mzexwkuefhkppyx”.
