# Supplementary material for: Melatonin orchestrates mitochondrial fusion dynamics-mediated WNT/β-catenin signaling to promote dopaminergic neuronal differentiation of human iPS and nerve regeneration in a MPTP-induced mouse model of Parkinson’s disease
Source: Cell Death Discov. 2025 Dec 20;12:1. doi: 10.1038/s41420-025-02906-x (PMC12780243; doi:10.1038/s41420-025-02906-x)
Supplement: Supplementary file 1 — Supplementary Figure Legends [file 41420_2025_2906_MOESM1_ESM.docx]

**Figure S1.** MT regulates the differentiation potential of hiPSCs into three germ layers in vitro. **(A)** Immunofluorescence staining of pluripotency markers (Nanog, OCT4, SSEA3, TRA-1-81) in hiPSCs. Scale bars, 50 μm. **(B-D)** Clonogenic assays, formation efficiency and colony diameter statistics of MT-hiPSCs. Scale bars, 20 μm. **(E-G)** Expression of the three germ layer markers (Pax6, Gata6, EOMES) at mRNA level. **P* < 0.05, ***P* < 0.01, ****P* < 0.001 *vs.*NC.

**Figure S2.** MT promoted the neural differentiation potential of hiPSCs by regulating MT receptor MT1/2. **(A-D)** Expression of pluripotency marker (Nanog) and three germ layer markers (Pax6, Gata6, EOMES) was determined by qRT-PCR. **(E, F)** Immunofluorescence staining of NSC markers (SOX2, SOX1) and the midbrain neural marker (Nurr1). Scale bars, 50 μm. **(G)** The statistics of the relative fluorescence intensity in differentiated NSCs. **(H)** Flow cytometry analysis of NSC markers (Pax6, Nestin, SOX1, SOX2) and the midbrain neural marker (Nurr1). **(I)** CCK8 experiments. **P* < 0.05, ***P* < 0.01 *vs.* NC. #*P* < 0.05, ##*P* < 0.01 *vs.* MT 10μM.

**Figure S3.** MT regulates mitochondrial dynamic balance. **(A)** Expression of mitochondrial fusion and fission-related proteins was determined by qRT-PCR after 7 days of MT and Luzindole treatment in hiPSCs. **(B-G)** Expression of mitochondrial fusion and fission-related proteins was determined by qRT-PCR after 7 days of MT and Luzindole treatment in NSCs. **(H-K)** Immunofluorescence staining of the mitochondrial fusion proteins MFN2 and the statistics of the relative fluorescence intensity after 7 days of MT and Luzindole treatment in NSCs. Scale bars, 50 μm. **P* < 0.05, ***P* < 0.01, ****P* < 0.001 *vs.* NC/NC(NSC). #*P* < 0.05, ##*P* < 0.01 *vs.* NSC MT 10μM/MT 10μM.

**Figure S4.** MT promoted the DAPCs differentiation potential of hiPSCs. **(A)** Heat map of upregulated and downregulated genes NSCs and DAPCs before and after 10μM MT treatment (n = 3).

**(B-E)** Gene ontology (GO) and Gene Set Enrichment Analysis (GSEA) from RNA-seq between NSCs and DAPCs. **(F-H)** GO and GSEA analysis from RNA-seq between DAPCs MT 0μM and DAPCs MT 10μM.
