# Supplementary figures and images for: Melatonin orchestrates mitochondrial fusion dynamics-mediated WNT/β-catenin signaling to promote dopaminergic neuronal differentiation of human iPS and nerve regeneration in a MPTP-induced mouse model of Parkinson’s disease
Source: Cell Death Discov. 2025 Dec 20;12:1. doi: 10.1038/s41420-025-02906-x (PMC12780243; doi:10.1038/s41420-025-02906-x)

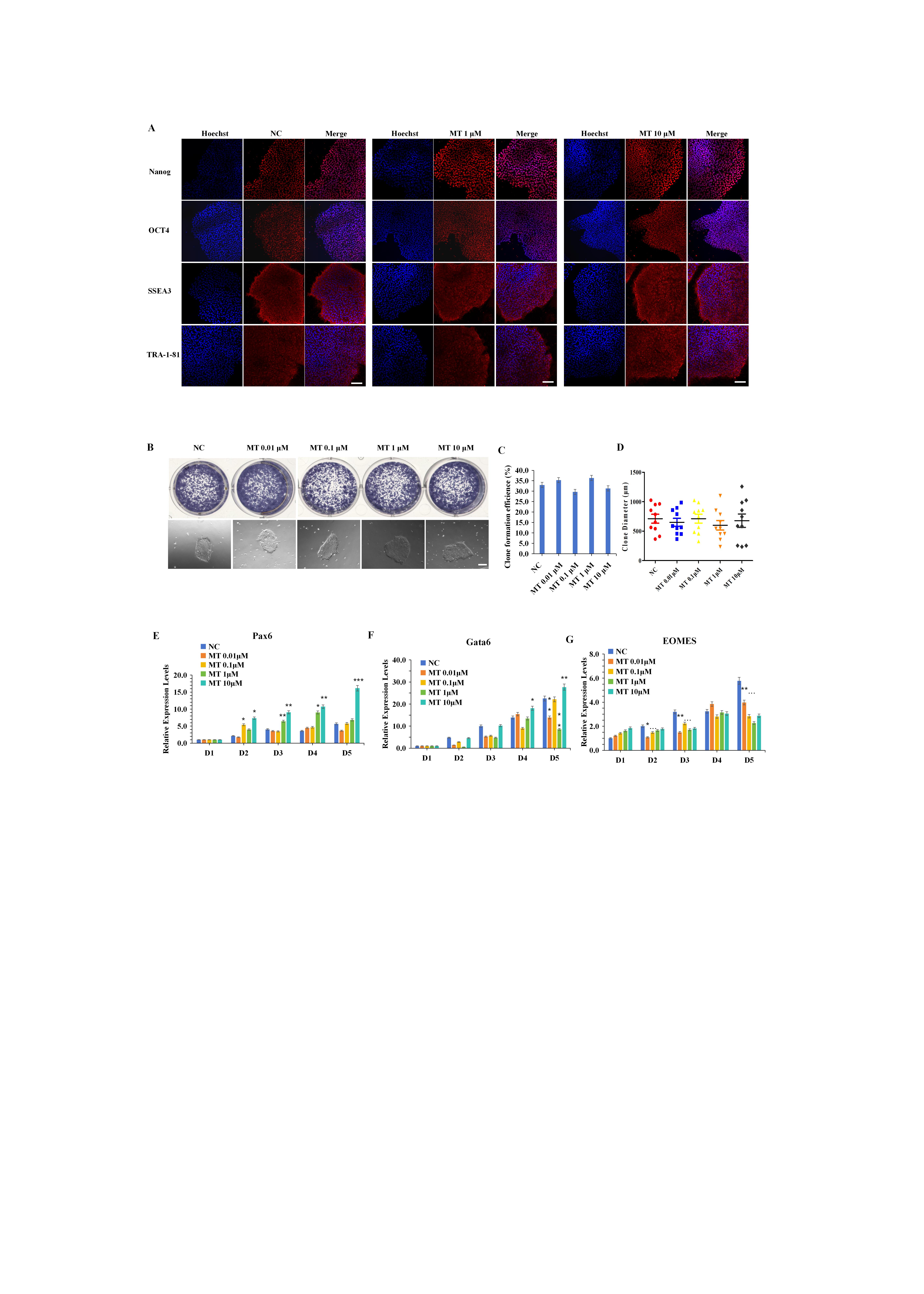

Supplement: Supplementary file 2 — Supplementary Figure 1 [file 41420_2025_2906_MOESM2_ESM.jpg]

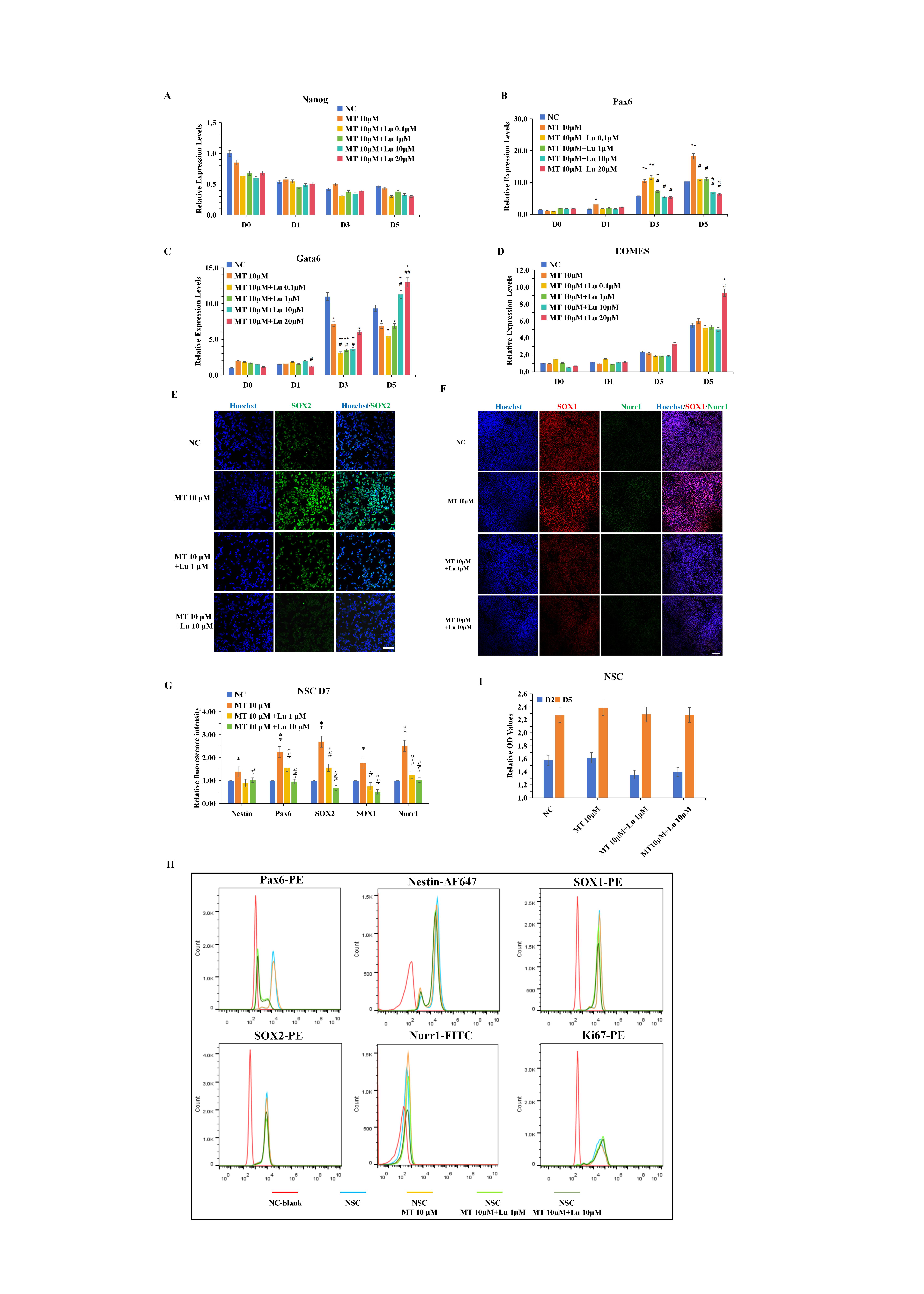

Supplement: Supplementary file 3 — Supplementary Figure 2 [file 41420_2025_2906_MOESM3_ESM.jpg]

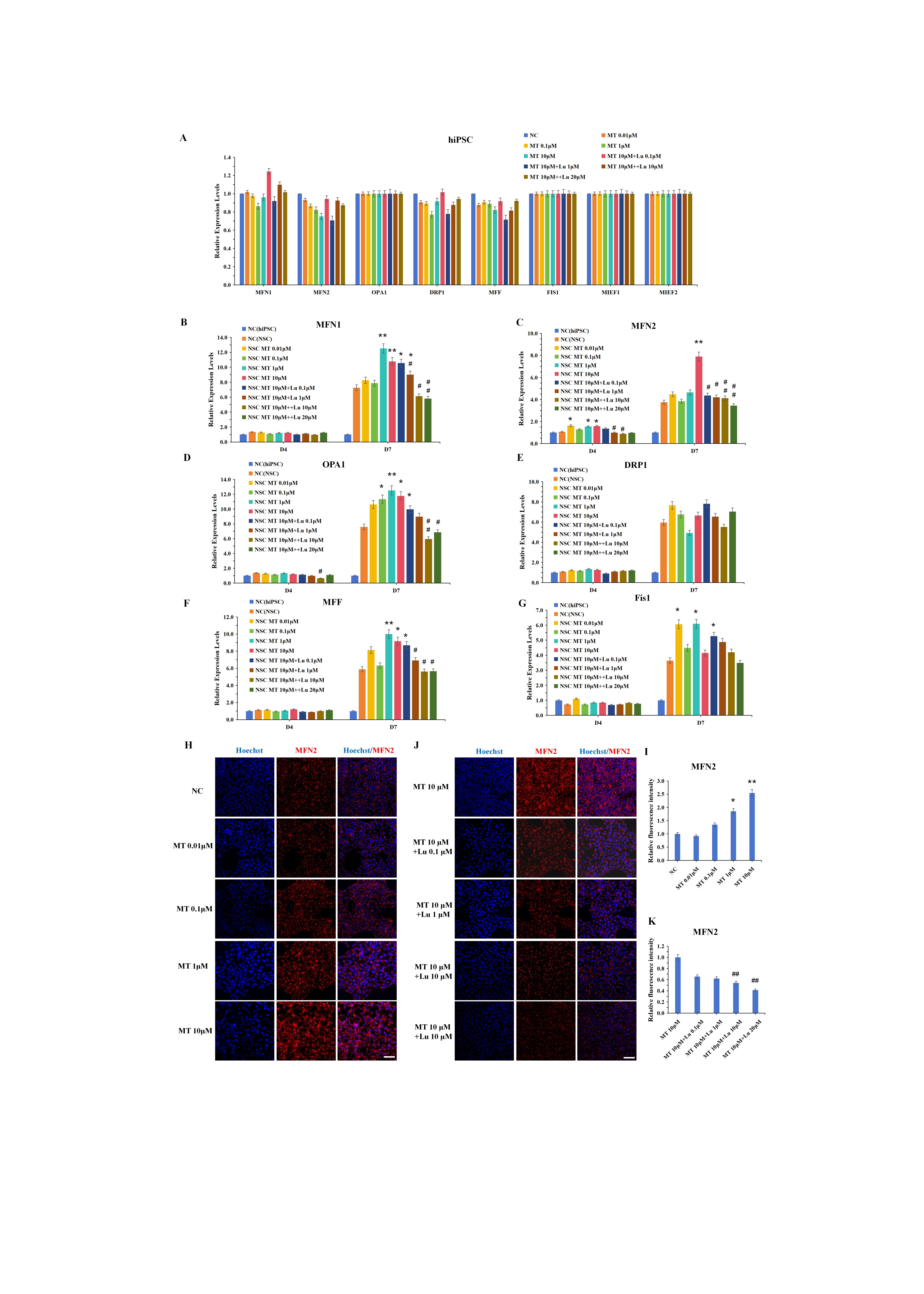

Supplement: Supplementary file 4 — Supplementary Figure 3 [file 41420_2025_2906_MOESM4_ESM.jpg]

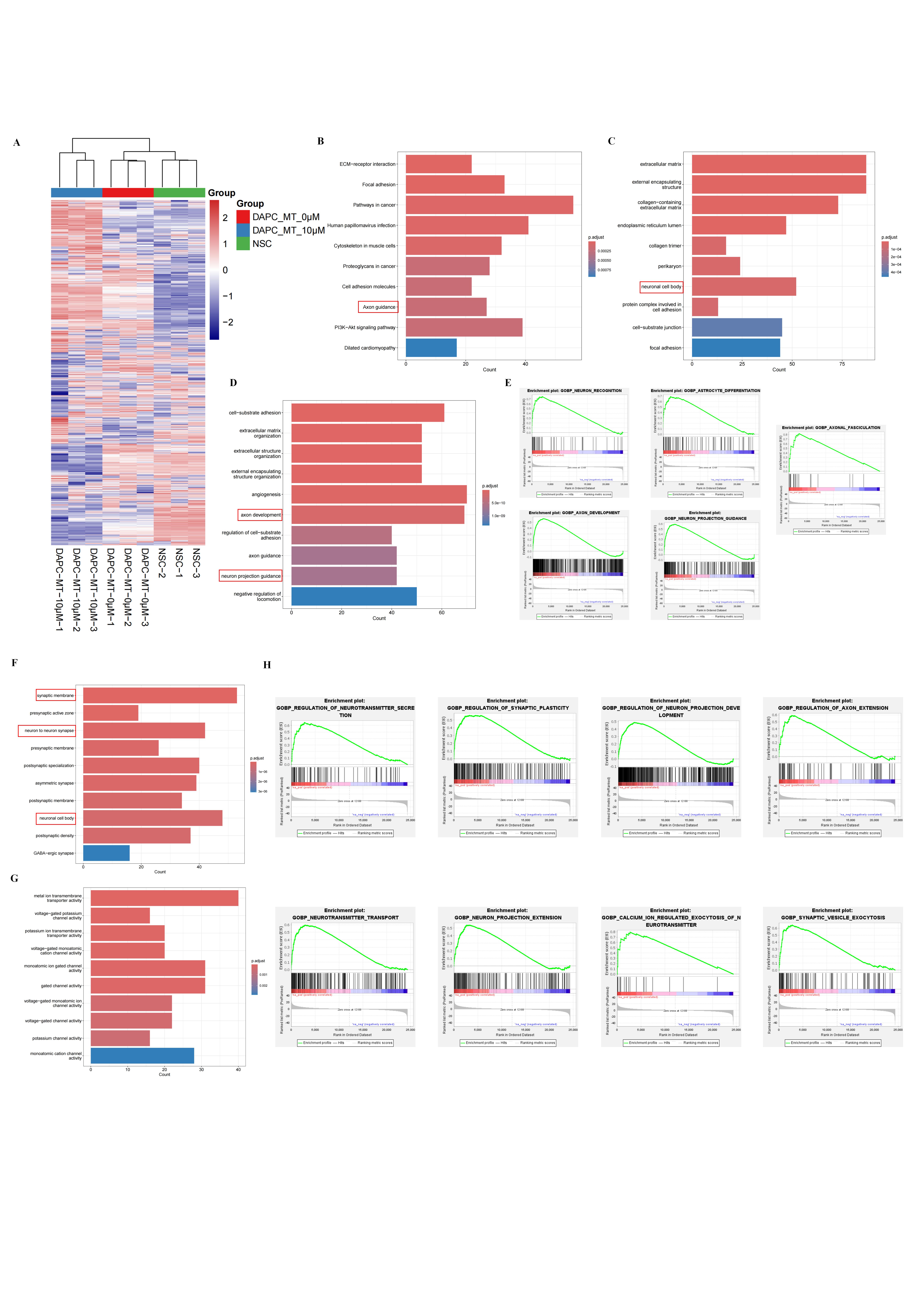

Supplement: Supplementary file 5 — Supplementary Figure 4 [file 41420_2025_2906_MOESM5_ESM.jpg]

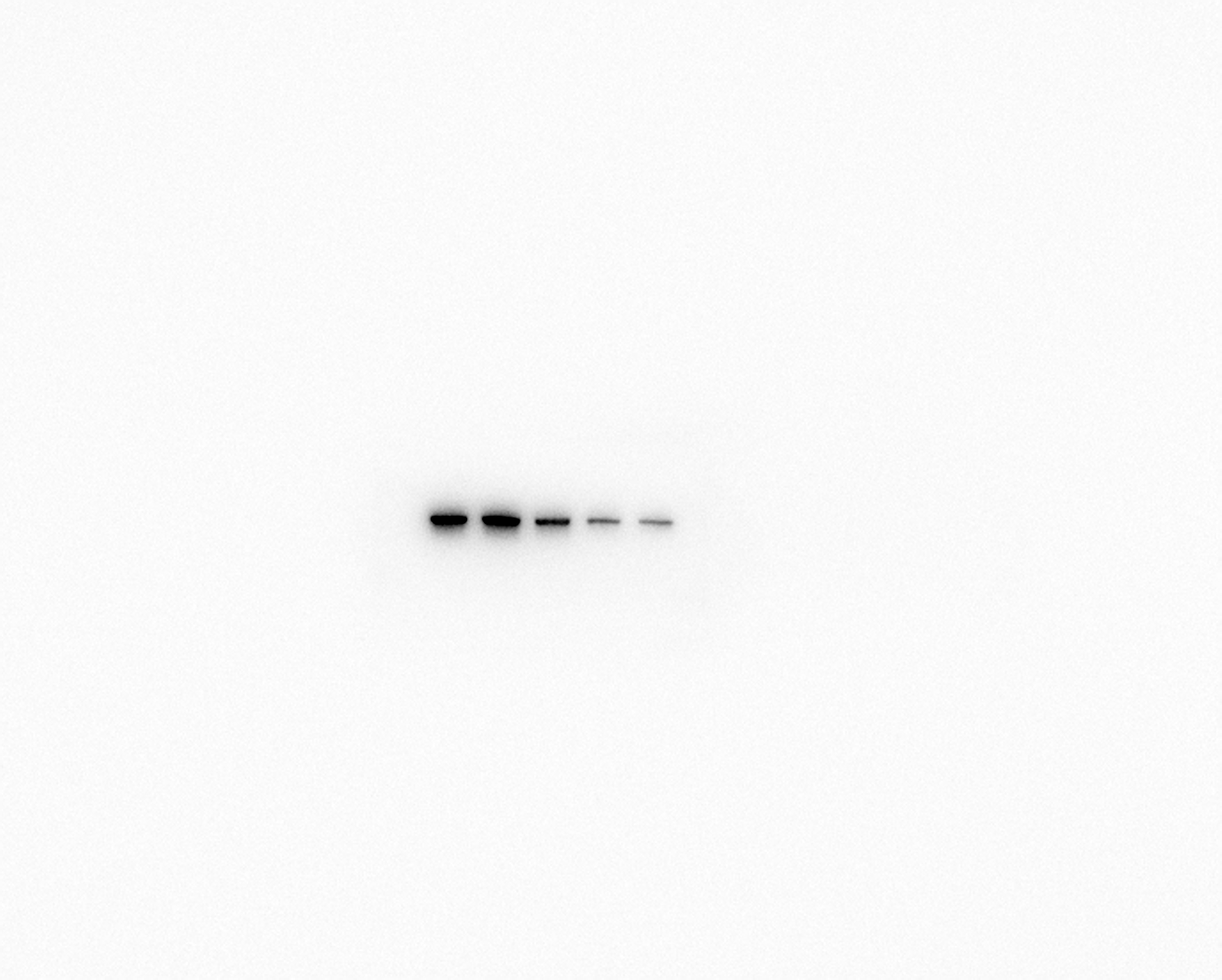

Supplement: Supplementary file 6 — Western blot_1 [file 41420_2025_2906_MOESM6_ESM.zip › Figure 3A/Figure3A MT1.Tif]

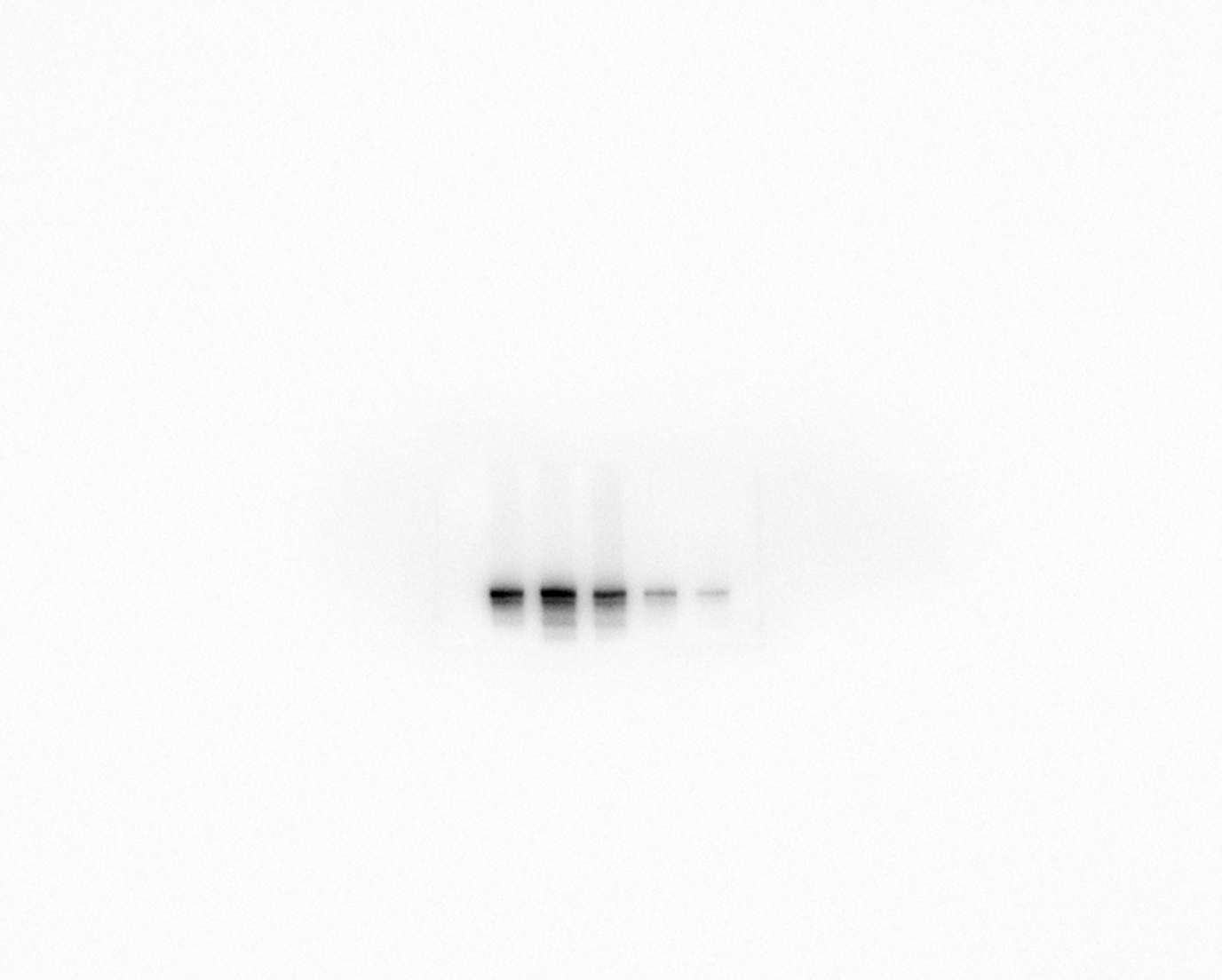

Supplement: Supplementary file 6 — Western blot_1 [file 41420_2025_2906_MOESM6_ESM.zip › Figure 3A/Figure3A MT2.Tif]

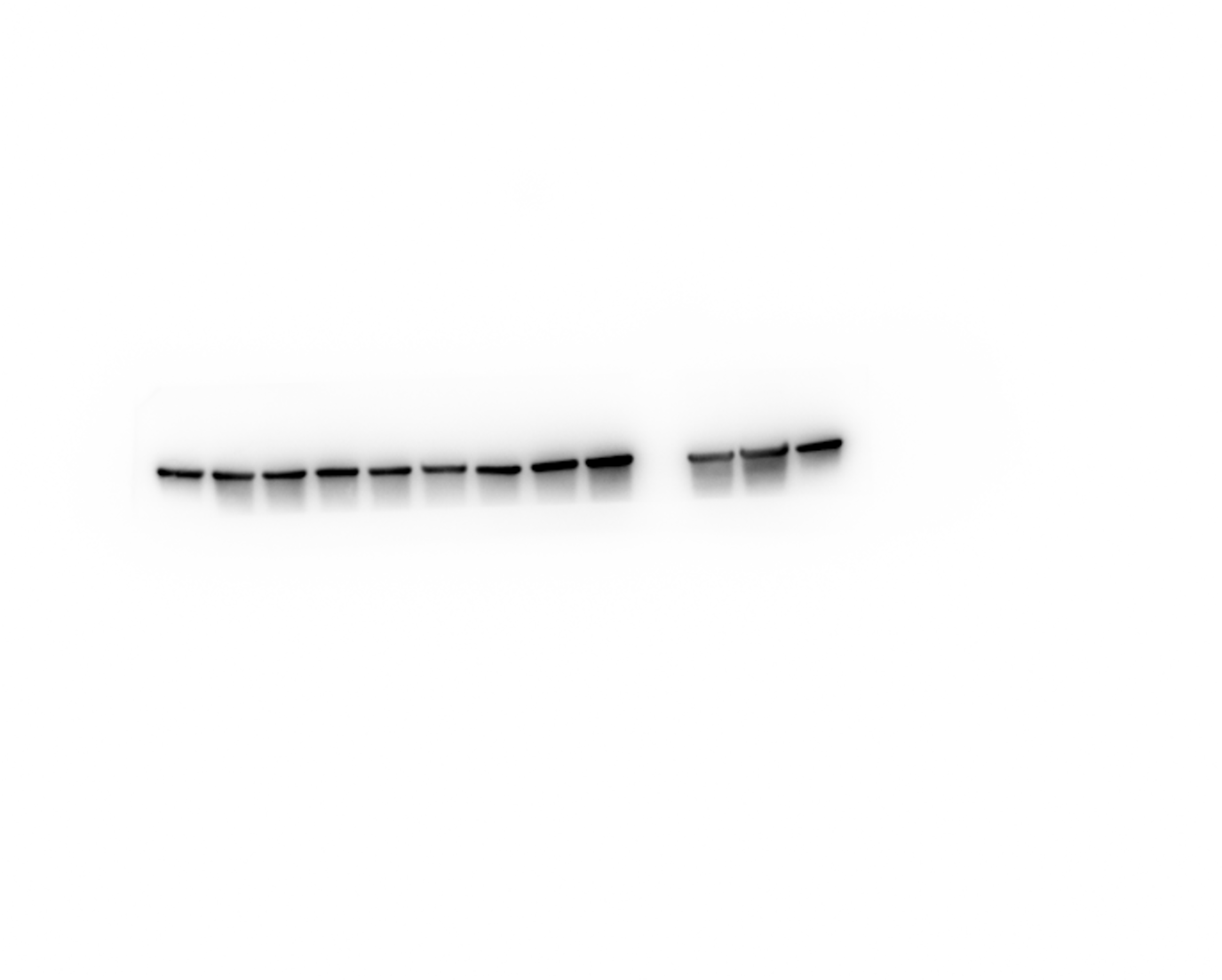

Supplement: Supplementary file 6 — Western blot_1 [file 41420_2025_2906_MOESM6_ESM.zip › Figure 3A/Figure3A a┬-Actin line 1-5.Tif]

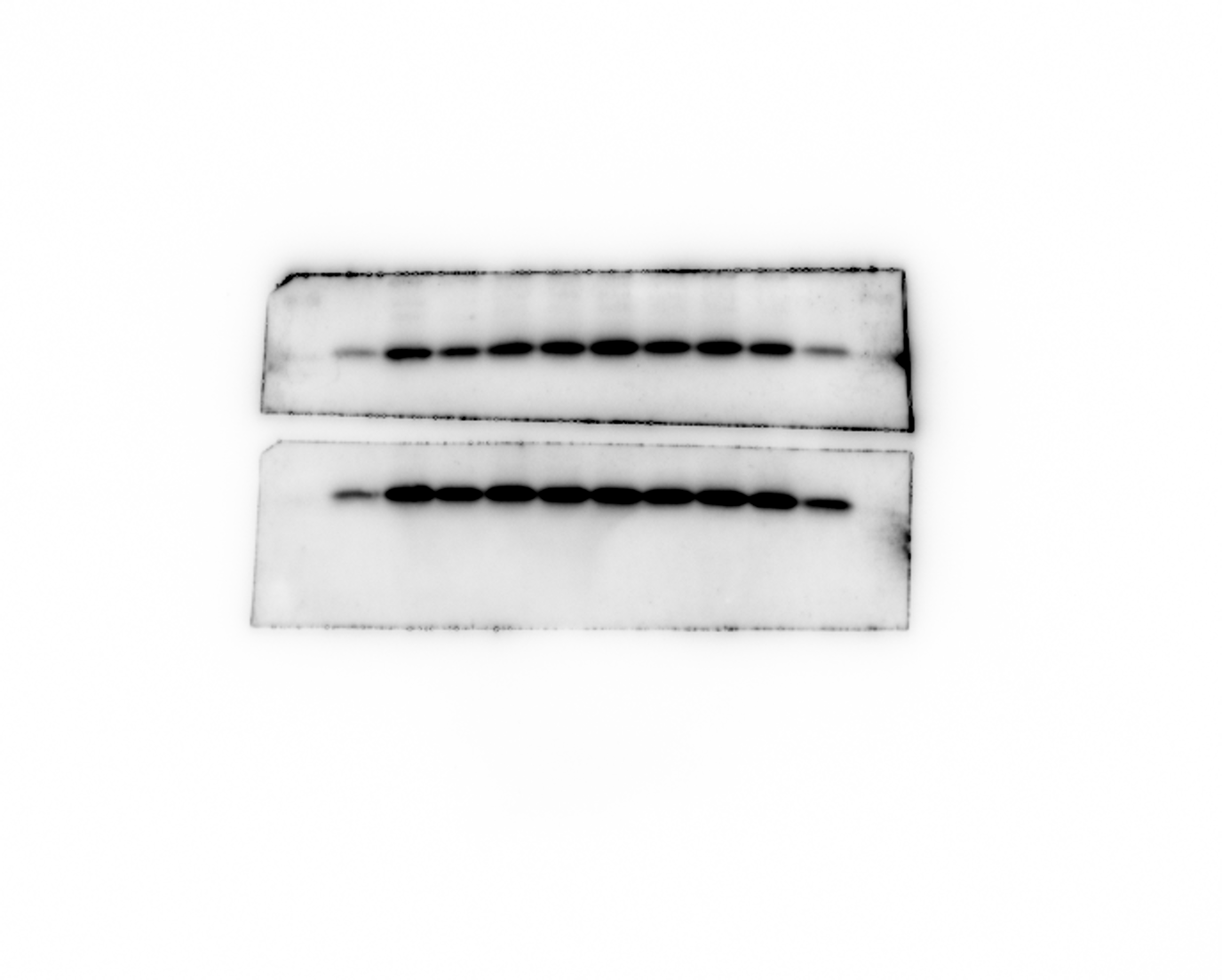

Supplement: Supplementary file 6 — Western blot_1 [file 41420_2025_2906_MOESM6_ESM.zip › Figure 4A/Figure 4A down-DRP1.Tif]

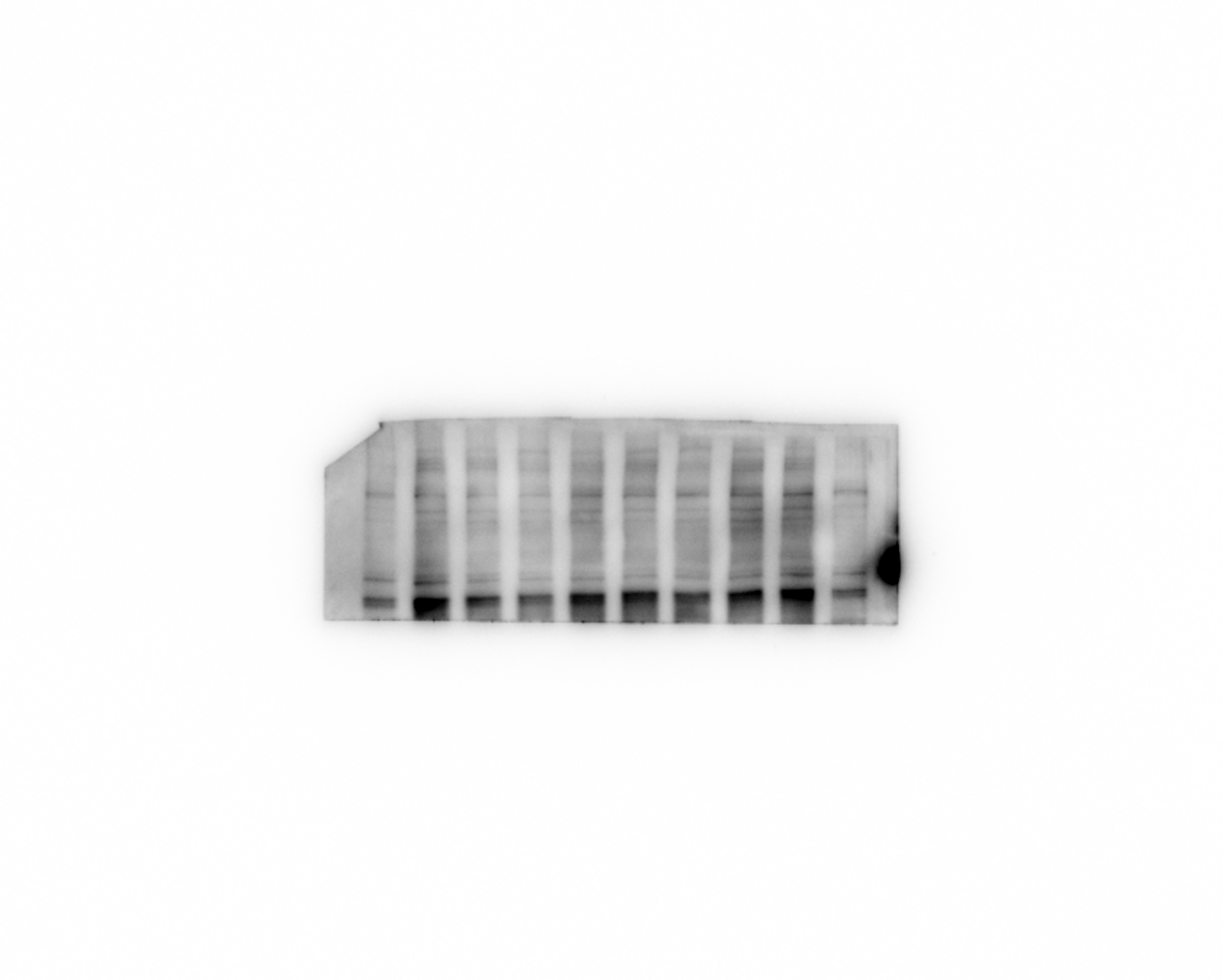

Supplement: Supplementary file 6 — Western blot_1 [file 41420_2025_2906_MOESM6_ESM.zip › Figure 4A/Figure 4A MFF.Tif]

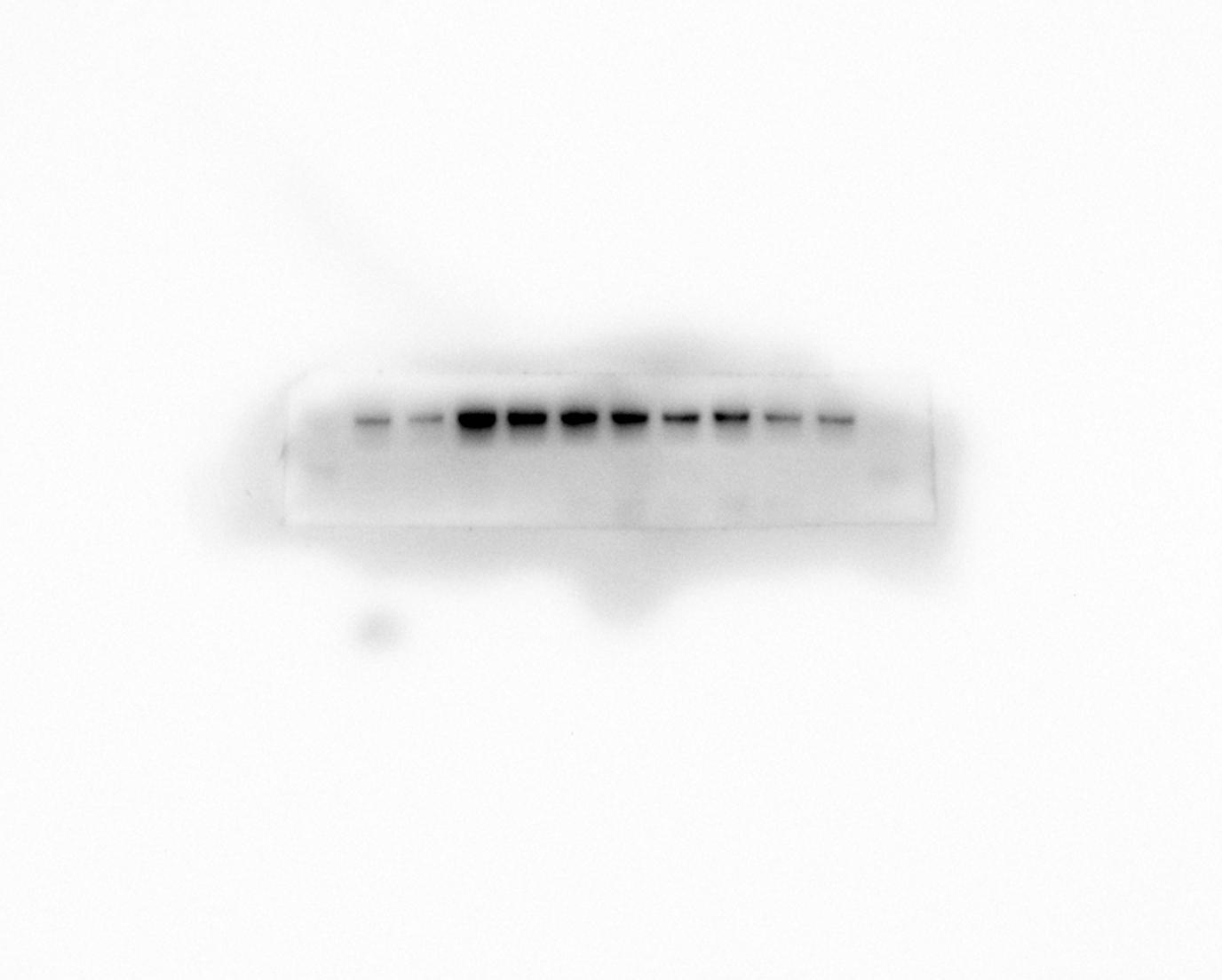

Supplement: Supplementary file 6 — Western blot_1 [file 41420_2025_2906_MOESM6_ESM.zip › Figure 4A/Figure 4A MFN1.Tif]

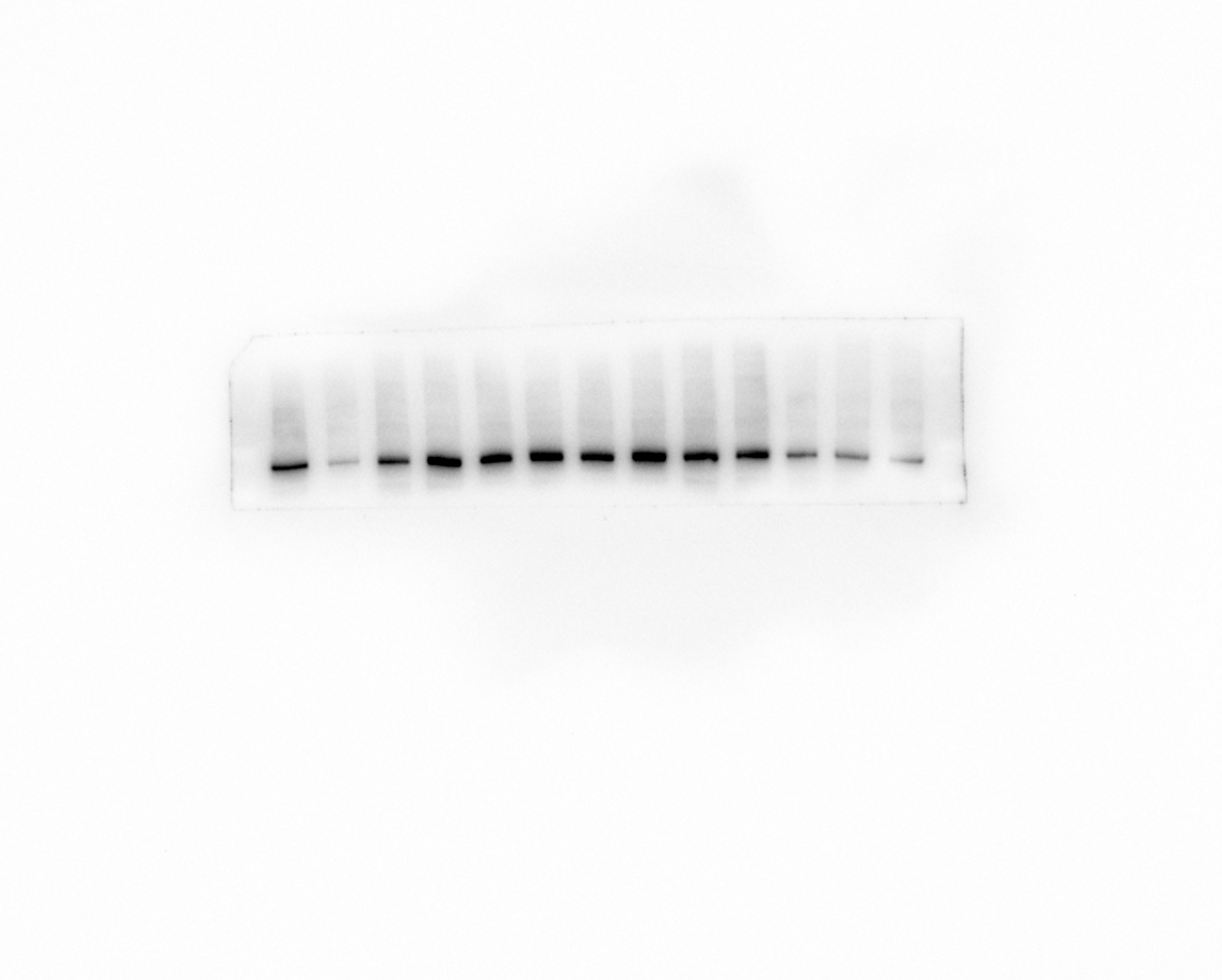

Supplement: Supplementary file 6 — Western blot_1 [file 41420_2025_2906_MOESM6_ESM.zip › Figure 4A/Figure 4A MFN2 line 2-11 10S.Tif]

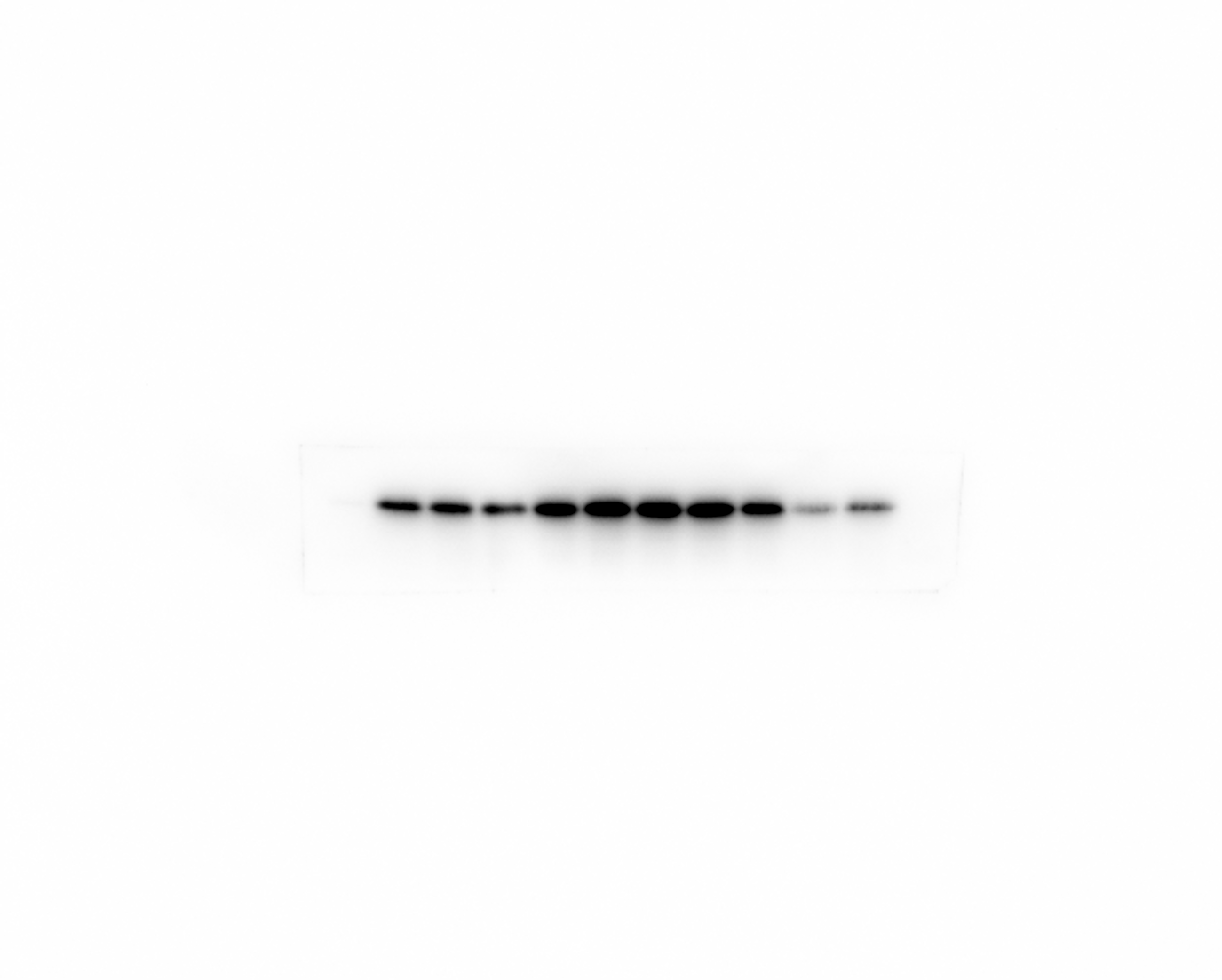

Supplement: Supplementary file 6 — Western blot_1 [file 41420_2025_2906_MOESM6_ESM.zip › Figure 4A/Figure 4A OPA1.Tif]

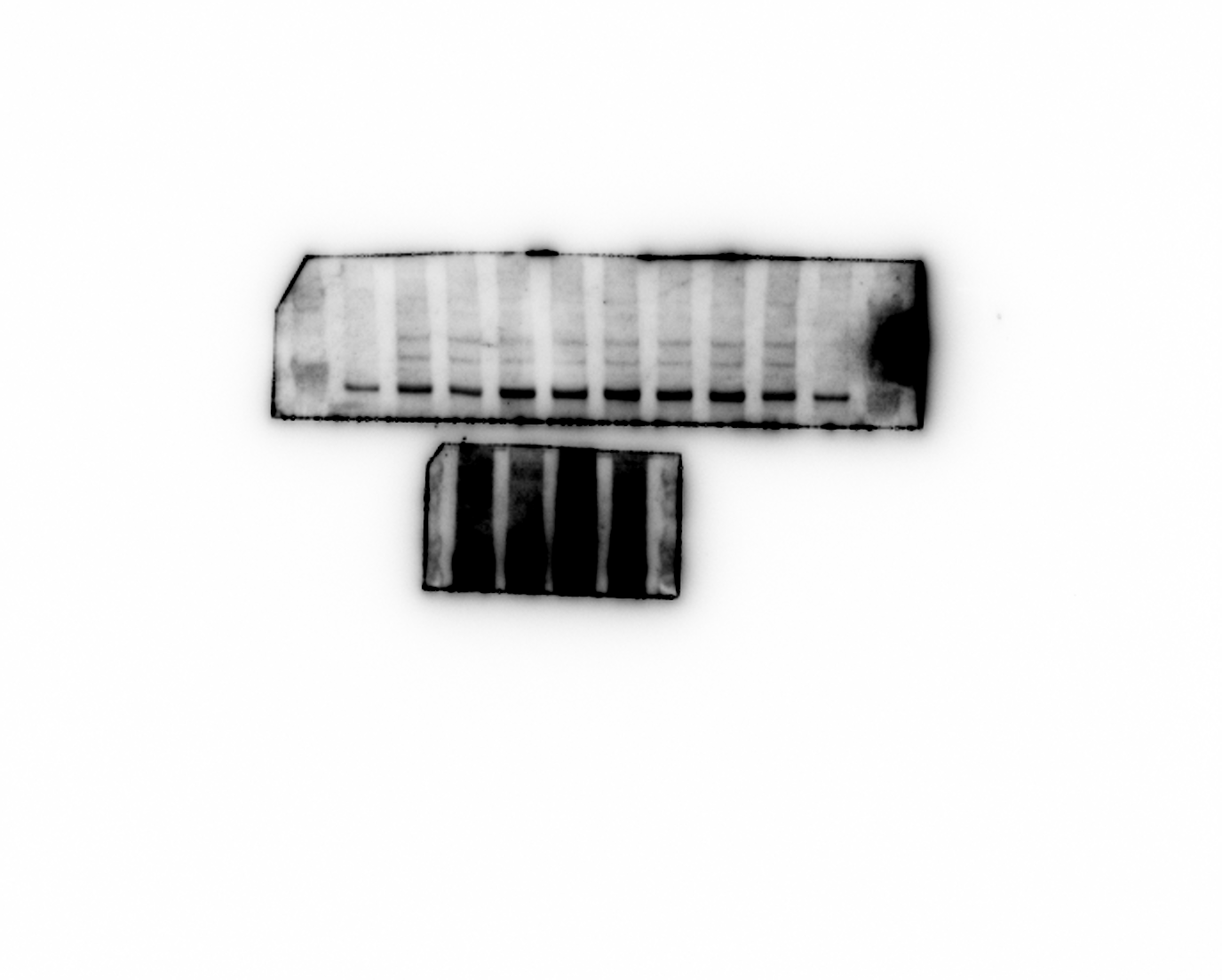

Supplement: Supplementary file 6 — Western blot_1 [file 41420_2025_2906_MOESM6_ESM.zip › Figure 4A/Figure 4A Up-Fis1.Tif]

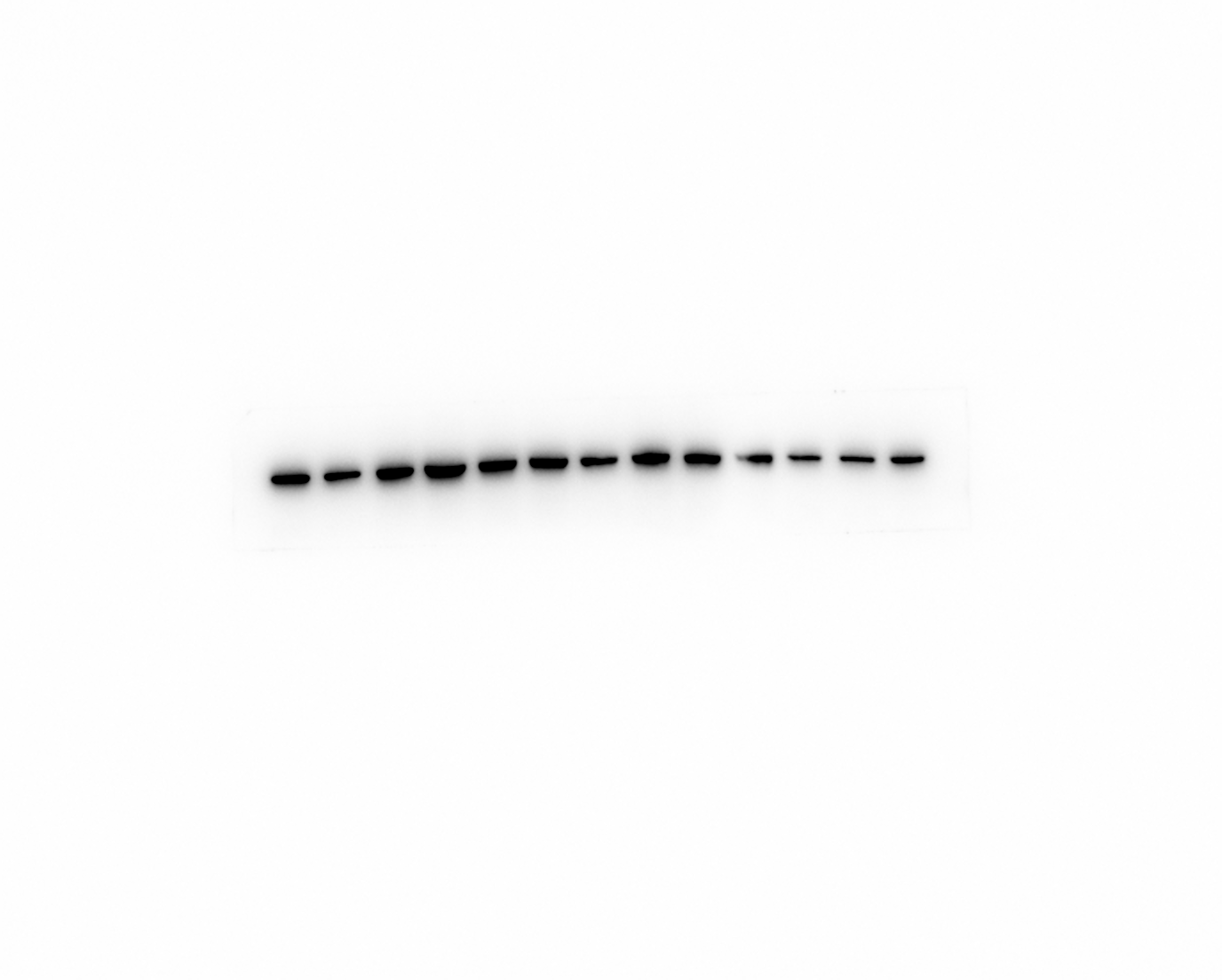

Supplement: Supplementary file 6 — Western blot_1 [file 41420_2025_2906_MOESM6_ESM.zip › Figure 4A/Figure 4A a┬-Actin line 1-10.Tif]

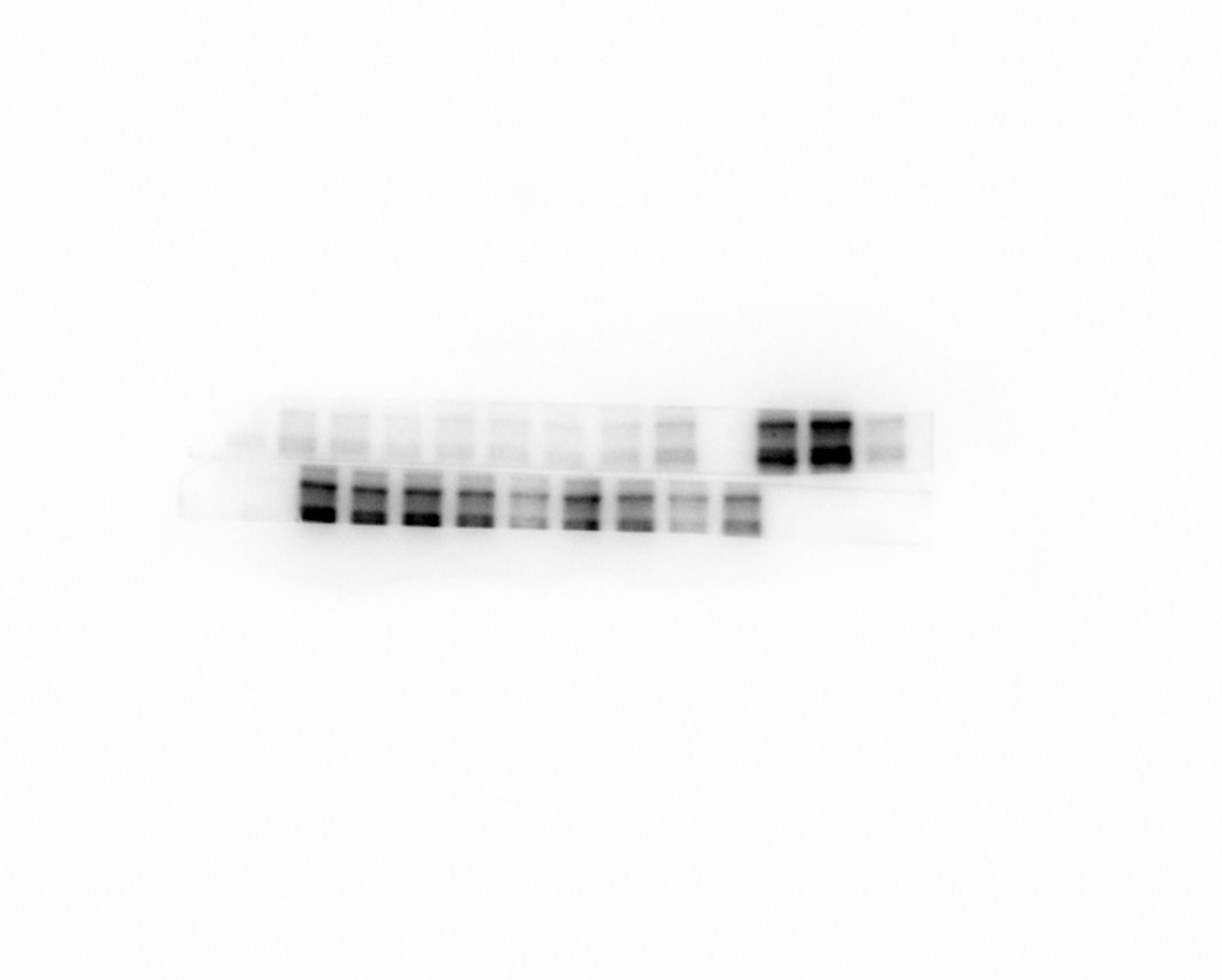

Supplement: Supplementary file 6 — Western blot_1 [file 41420_2025_2906_MOESM6_ESM.zip › Figure 4F/Figure 4F MFN2 up line11-13.Tif]

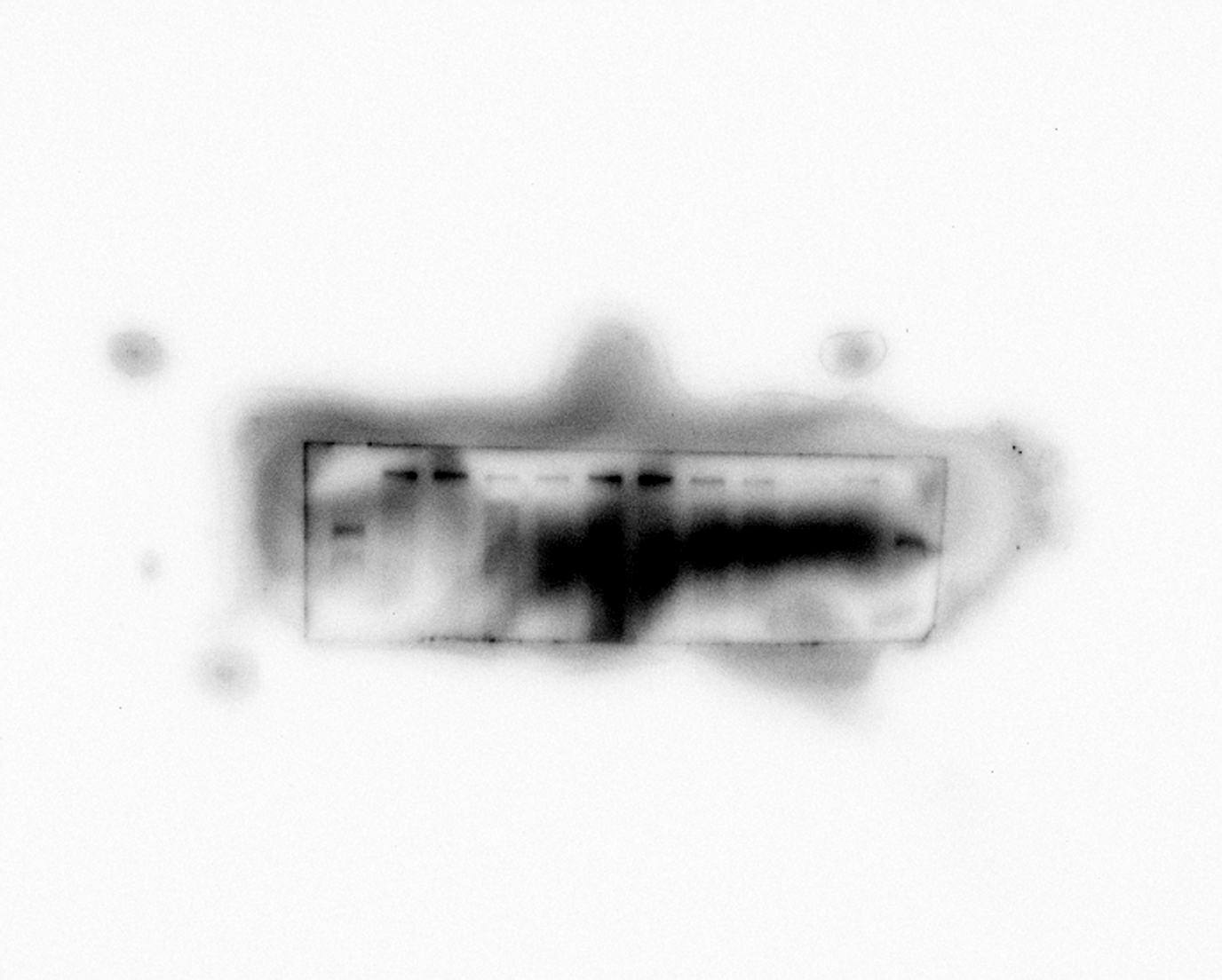

Supplement: Supplementary file 6 — Western blot_1 [file 41420_2025_2906_MOESM6_ESM.zip › Figure 5A/Figure 5A a┬-Catenin line 6-8.Tif]

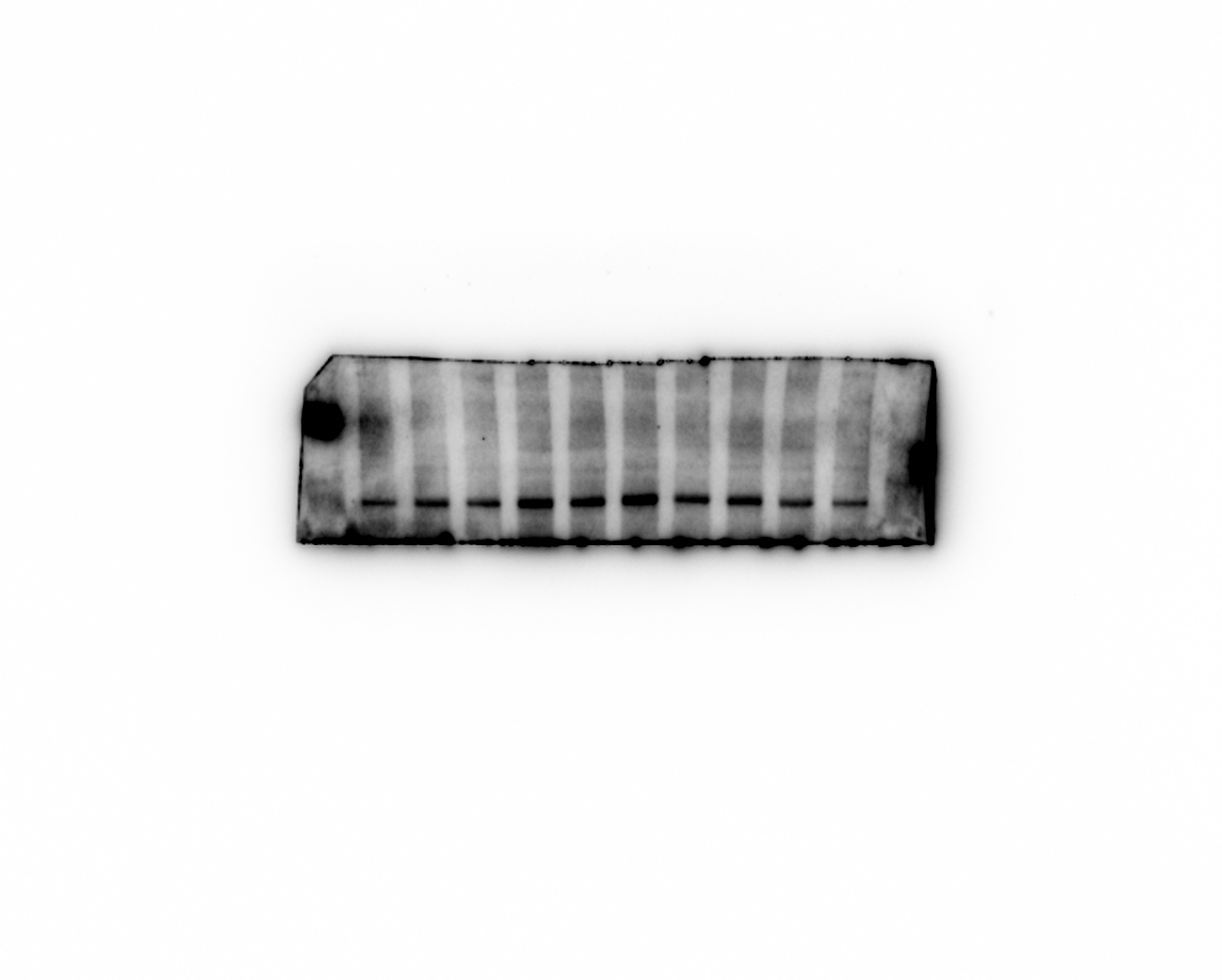

Supplement: Supplementary file 6 — Western blot_1 [file 41420_2025_2906_MOESM6_ESM.zip › Figure 5K/Figure 5K Wnt3a.Tif]

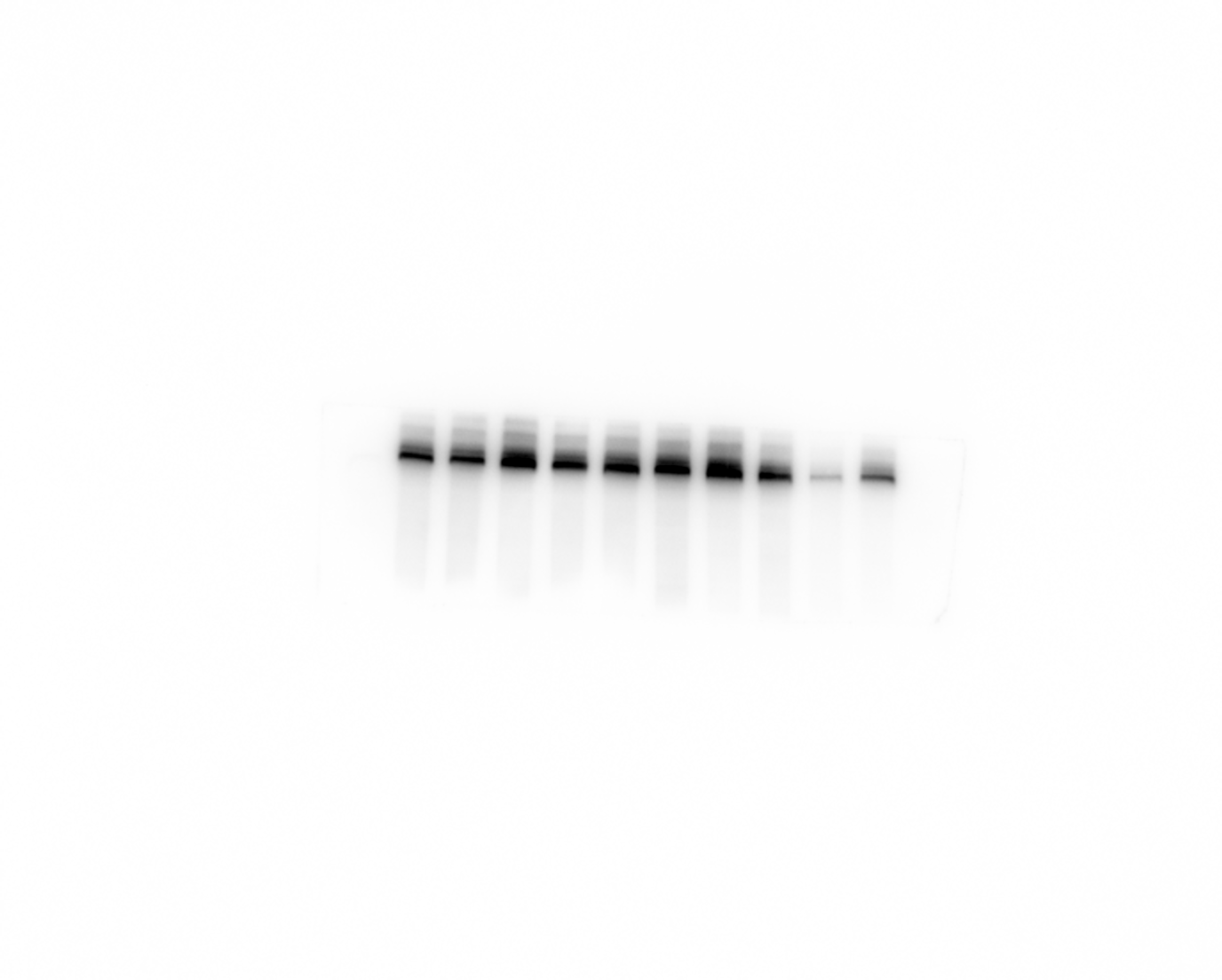

Supplement: Supplementary file 6 — Western blot_1 [file 41420_2025_2906_MOESM6_ESM.zip › Figure 5K/Figure 5K Wnt5a.Tif]

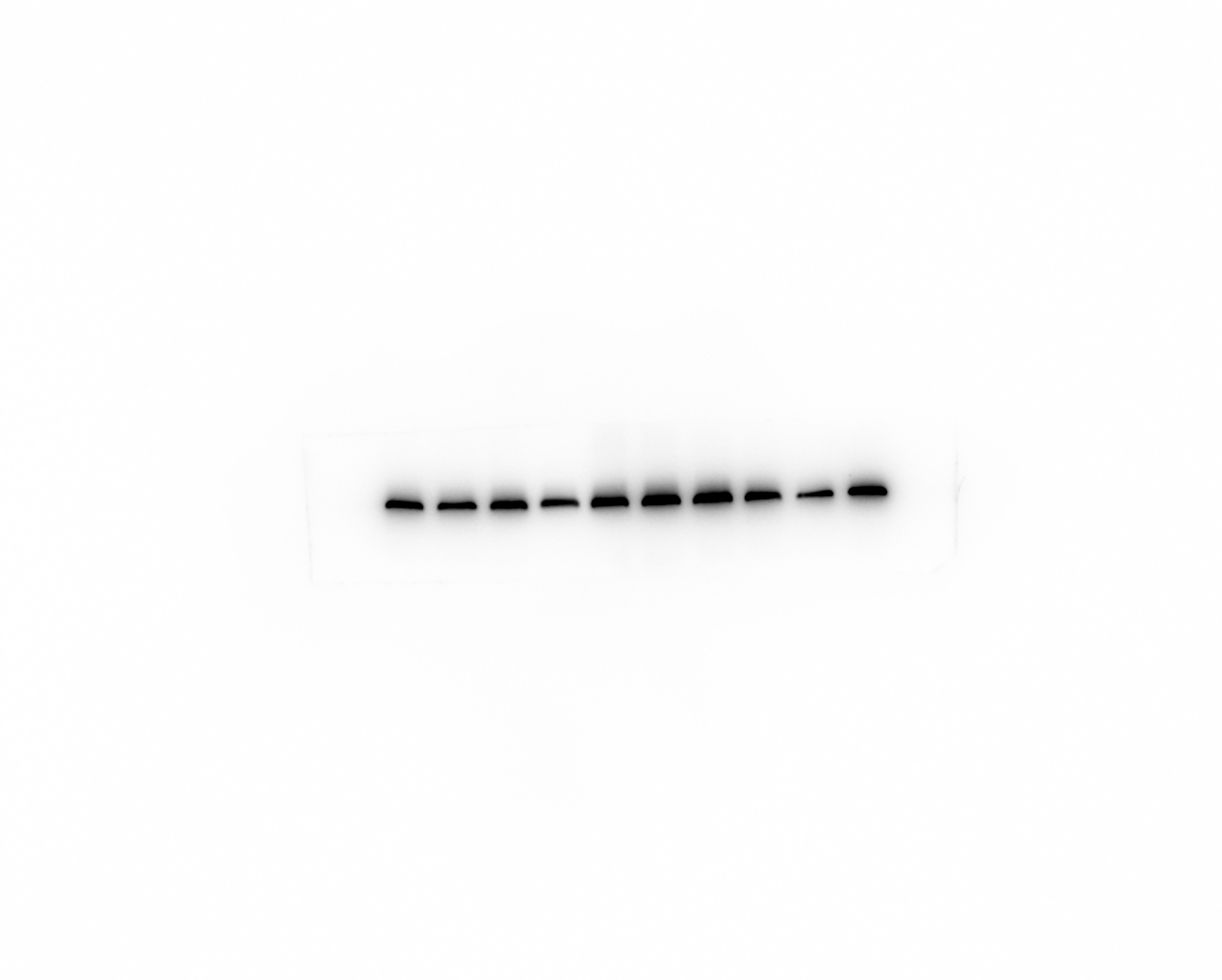

Supplement: Supplementary file 6 — Western blot_1 [file 41420_2025_2906_MOESM6_ESM.zip › Figure 5K/Figure 5K a┬-Actin.Tif]

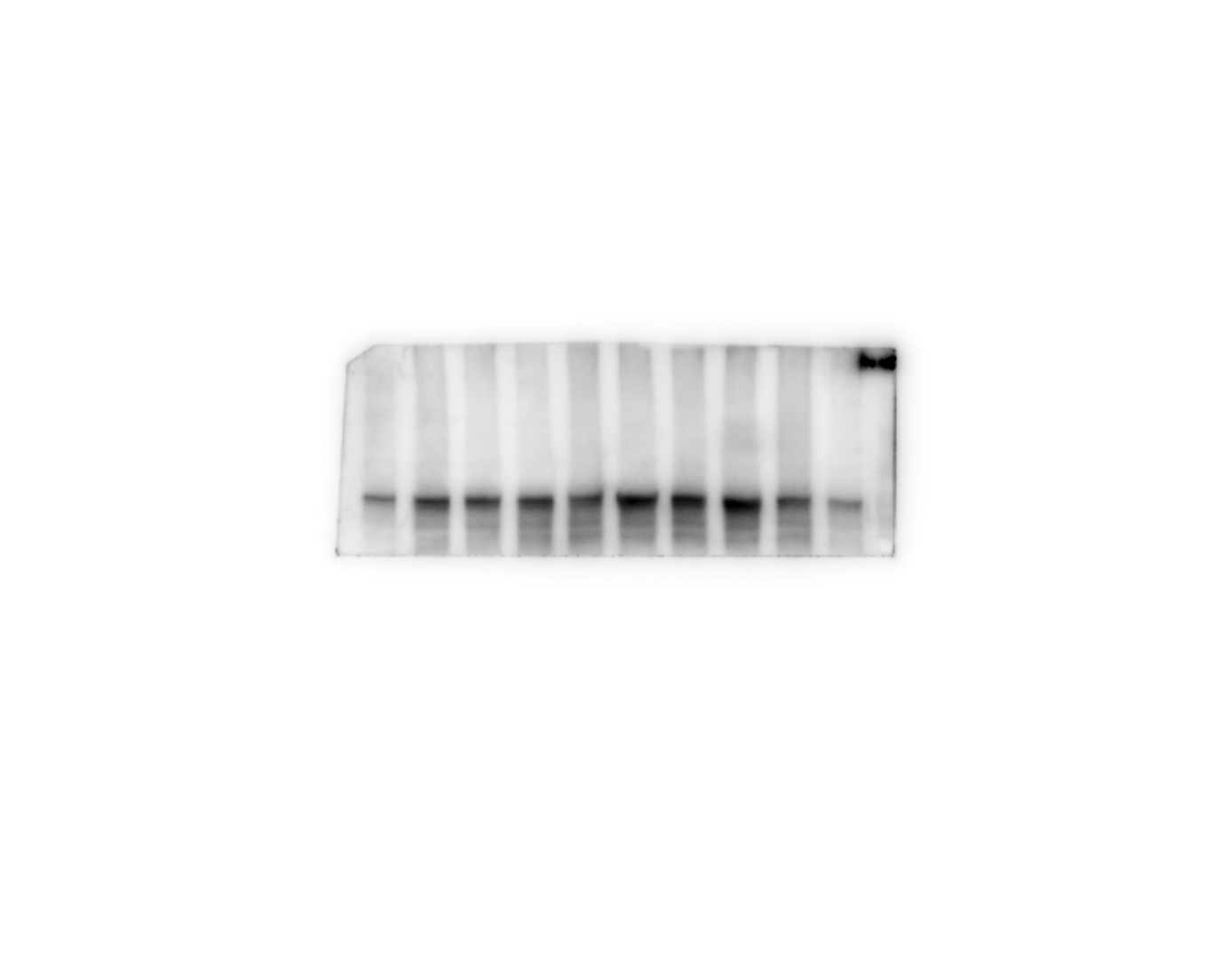

Supplement: Supplementary file 6 — Western blot_1 [file 41420_2025_2906_MOESM6_ESM.zip › Figure 5K/Figure 5K a┬-Catenin.Tif]
