# Supplementary material for: Melatonin orchestrates mitochondrial fusion dynamics-mediated WNT/β-catenin signaling to promote dopaminergic neuronal differentiation of human iPS and nerve regeneration in a MPTP-induced mouse model of Parkinson’s disease
Source: Cell Death Discov. 2025 Dec 20;12:1. doi: 10.1038/s41420-025-02906-x (PMC12780243; doi:10.1038/s41420-025-02906-x)
Supplement: Supplementary file 7 — Supplementary Table 1 [file 41420_2025_2906_MOESM7_ESM.docx]

**Supplementary Table 1**

**Primer sequence for RT-qPCR**

| *GENE* | Forward primers (5’-3’) | Reverse primers (5’-3’) |
| --- | --- | --- |
| *β-actin* | AAACTGGAACGGTGAAGGTG | AGTGGGGTGGCTTTTAGGAT |
| *Nanog* | CAGCCCCGATTCTTCCACCAGTCCC | CGGAAGATTCCCAGTCGGGTTCACC |
| *OCT4* | GACAGGGGGAGGGGAGGAGCTAGG | CTTCCCTCCAACCAGTTGCCCCAAAC |
| *Pax6* | AGTGCCCGTCCATCTTTGC | CGCTTGGTATGTTATCGTTGGT |
| *Nestin* | GAAGGGCAATCACAACAGGTG | GGGGCCACATCATCTTCCA |
| *Gata6* | ACCACCTTATGGCGCAGAAA | ATAGCAAGTGGTCTGGGCAC |
| *EOMES* | AAGGGGAGAGTTTCATCATCCC | GGCGCAAGAAGAGGATGAAATAG |
| *MFN1* | ATGACCTGGTGTTAGTAGACAGT | AGACATCAGCATCTAGGCAAAAC |
| *MFN2* | CTCTCGATGCAACTCTATCGTC | TCCTGTACGTGTCTTCAAGGAA |
| *OPA1* | CCGTTAGCCCTGAGACCATA | AAGTCAACAAGCACCATCCT |
| *DRP1* | TTTGACACTTGTGGATTTGCCA | AGTGACAGCGAGGATAATGGA |
| *MFF* | ACTGAAGGCATTAGTCAGCGA | TCCTGCTACAACAATCCTCTCC |
| *FIS1* | AGCGGGATTACGTCTTCTACC | CATGCCCACGAGTCCATCTTT |
| *MIEF1* | CCTAAACCCCAAGGACAAGG | TGGCTGTTGGGTCTGTTCTT |
| *MIEF2* | GAAGCGGTTCATTGACAGGG | GCTAAGGGCAGCAGGTGGAG |
| *b-Catenin* | CATCTACACAGTTTGATGCTGCT | GCAGTTTTGTCAGTTCAGGGA |
