# Supplementary material for: Melatonin orchestrates mitochondrial fusion dynamics-mediated WNT/β-catenin signaling to promote dopaminergic neuronal differentiation of human iPS and nerve regeneration in a MPTP-induced mouse model of Parkinson’s disease
Source: Cell Death Discov. 2025 Dec 20;12:1. doi: 10.1038/s41420-025-02906-x (PMC12780243; doi:10.1038/s41420-025-02906-x)
Supplement: Supplementary file 8 — Supplementary Table 2 [file 41420_2025_2906_MOESM8_ESM.docx]

**Supplementary Table 2**

**Antibodies used in immunofluorescence staining**

| Antibodies | Source | Dilution |
| --- | --- | --- |
| Nanog | Invitrogen, MA1-017 | 1:400 |
| OCT4 | Santa Cruz, sc-5279 | 1:400 |
| SSEA3 | Invitrogen, MA1-020X | 1:200 |
| TRA-1-81 | Invitrogen, MA1-024 | 1:200 |
| Pax6 | CST, 60433S | 1:200 |
| Nestin | CST, 33475S | 1:2000 |
| SOX1 | Invitrogen, MA5-32447 | 1:200 |
| SOX2 | Invitrogen, MA1-014 | 1:200 |
| Nurr1 | Santa Cruz, sc-990 | 1:100 |
| TH | Santa Cruz, sc-25269 | 1:100 |
| EN1 | Santa Cruz, sc-66876 | 1:100 |
| NFL | Santa Cruz, sc-58559 | 1:200 |
| NeuN | CST, 24307S | 1:100 |
| MFN2 | CST, 9482S | 1:50 |
| β-catenin | CST, 8480S | 1:100 |
| Nuclei | Invitrogen, H3570 | 1:1000 |
