# Supplementary material for: Melatonin orchestrates mitochondrial fusion dynamics-mediated WNT/β-catenin signaling to promote dopaminergic neuronal differentiation of human iPS and nerve regeneration in a MPTP-induced mouse model of Parkinson’s disease
Source: Cell Death Discov. 2025 Dec 20;12:1. doi: 10.1038/s41420-025-02906-x (PMC12780243; doi:10.1038/s41420-025-02906-x)
Supplement: Supplementary file 9 — Supplementary Table 3 [file 41420_2025_2906_MOESM9_ESM.docx]

**Supplementary Table 3**

**Antibodies used in Flow Cytometry**

| Antibodies | Source | Dilution |
| --- | --- | --- |
| Nestin(10c2) Alexa Fluor^®^647 | Santa Cruz, sc-23927 AF647 | 1:20 |
| PharmingenTM PE Mouse anti-Human Pax6 | BD, 561552 | 1:20 |
| PharmingenTM PE Mouse anti-Human SOX1 | BD, 561592 | 1:20 |
| PharmingenTM PE Mouse anti-Human SOX2 | BD, 560291 | 1:20 |
| Nurr1 (F5) FITC | Santa Cruz, sc-376984 FITC | 1:20 |
| PE anti-huamn Ki67 | BioLegend, 350504 | 1:20 |
