# Supplementary material for: Melatonin orchestrates mitochondrial fusion dynamics-mediated WNT/β-catenin signaling to promote dopaminergic neuronal differentiation of human iPS and nerve regeneration in a MPTP-induced mouse model of Parkinson’s disease
Source: Cell Death Discov. 2025 Dec 20;12:1. doi: 10.1038/s41420-025-02906-x (PMC12780243; doi:10.1038/s41420-025-02906-x)
Supplement: Supplementary file 10 — Supplementary Table 4 [file 41420_2025_2906_MOESM10_ESM.docx]

**Supplementary Table 4**

**Antibodies used in Western Blotting**

| Antibodies | Source | Dilution |
| --- | --- | --- |
| β-actin | Invitrogen, MA1-140 | 1:1000 |
| MFN1 | Invitrogen, MA5-32496 | 1:1000 |
| MFN2 | CST, 9482S | 1:1000 |
| Fis1 | Invitrogen, PA5-22142 | 1:1000 |
| DRP1 | Invitrogen, MA5-38045 | 1:1000 |
| OPA1 | Invitrogen, PA5-57874 | 1:1000 |
| MFF | Invitrogen, PA5-52765 | 1:1000 |
| β-catenin | CST, 8480S | 1:1000 |
| WNT3a | Invitrogen, PA5-102317 | 1:1000 |
| WNT5a | Invitrogen, MA5-14946 | 1:1000 |
| MT1 | Invitrogen, PA5-75749 | 1:1000 |
| MT2 | Invitrogen, PA5-102107 | 1:1000 |
