# Supplementary material for: Melatonin orchestrates mitochondrial fusion dynamics-mediated WNT/β-catenin signaling to promote dopaminergic neuronal differentiation of human iPS and nerve regeneration in a MPTP-induced mouse model of Parkinson’s disease
Source: Cell Death Discov. 2025 Dec 20;12:1. doi: 10.1038/s41420-025-02906-x (PMC12780243; doi:10.1038/s41420-025-02906-x)
Supplement: Supplementary file 6 — Western blot_1 [file 41420_2025_2906_MOESM6_ESM.zip › Western blot.doc]

**Western blot**

**Figure 3A**

**Figure3A MT1**

**Figure3A MT2**

**Figure3A β-Actin line 1-5**

**Figure 4A**

**Figure 4A down-DRP1**

**Figure 4A MFF**

**Figure 4A MFN1**

**Figure 4A MFN2 line 2-11**

**Figure 4A OPA1**

**Figure 4A Up-Fis1**

**Figure 4A β-Actin line 1-10**

**Figure 4F**

**Figure 4F MFN2 up line11-13**

**Figure 4F β-Actin line 11-13**

**Figure 5A**

**Figure 5A β-Actin line 11-13**

**Figure 5A β-Catenin line 6-8**

**Figure 5K**

**Figure 5K Wnt3a**

**Figure 5K Wnt5a**

**Figure 5K β-Actin**

**Figure 5K β-Catenin**
